# Genome-Wide Identification and Characterization of *RdHSP* Genes Related to High Temperature in *Rhododendron delavayi*

**DOI:** 10.3390/plants13131878

**Published:** 2024-07-07

**Authors:** Cheng Wang, Xiaojing Wang, Ping Zhou, Changchun Li

**Affiliations:** 1 Hubei Key Laboratory of Quality Control of Characteristic Fruits and Vegetables, College of Life Science and Technology, Hubei Engineering University, Xiaogan 432000, China; chengwang@hbeu.edu.cn; 2 Hubei Province Research Center of Engineering Technology for Utilization of Botanical Functional Ingredients, Xiaogan 432000, China; 3 The Key Laboratory of Plant Resources Conservation and Germplasm Innovation in the Mountainous Region (Ministry of Education), College of Life Sciences, Guizhou University, Guiyang 550025, China; xjwang8@gzu.edu.cn (X.W.); m17855848205@gmail.com (P.Z.)

**Keywords:** *RdHSP* gene family, expression pattern, high-temperature stress, RNA-seq data, subcellular localization

## Abstract

Heat shock proteins (HSPs) are molecular chaperones that play essential roles in plant development and in response to various environmental stresses. Understanding *R. delavayi HSP* genes is of great importance since *R. delavayi* is severely affected by heat stress. In the present study, a total of 76 *RdHSP* genes were identified in the *R. delavayi* genome, which were divided into five subfamilies based on molecular weight and domain composition. Analyses of the chromosome distribution, gene structure, and conserved motif of the *RdHSP* family genes were conducted using bioinformatics analysis methods. Gene duplication analysis showed that 15 and 8 *RdHSP* genes were obtained and retained from the WGD/segmental duplication and tandem duplication, respectively. *Cis*-element analysis revealed the importance of *RdHSP* genes in plant adaptations to the environment. Moreover, the expression patterns of *RdHSP* family genes were investigated in *R. delavayi* treated with high temperature based on our RNA-seq data, which were further verified by qRT-PCR. Further analysis revealed that nine candidate genes, including six *RdHSP20* subfamily genes (*RdHSP20.4*, *RdHSP20.8*, *RdHSP20.6*, *RdHSP20.3*, *RdHSP20.10*, and *RdHSP20.15*) and three *RdHSP70* subfamily genes (*RdHSP70.15*, *RdHSP70.21*, and *RdHSP70.16*), might be involved in enhancing the heat stress tolerance. The subcellular localization of two candidate *RdHSP* genes (*RdHSP20.8* and *RdHSP20.6*) showed that two candidate RdHSPs were expressed and function in the chloroplast and nucleus, respectively. These results provide a basis for the functional characterization of *HSP* genes and investigations on the molecular mechanisms of heat stress response in *R. delavayi*.

## 1. Introduction

During growth and development, the sessile nature of plants makes them vulnerable to various kinds of biotic (herbivore and pathogen attacks) and abiotic stresses (drought, flooding, salinity, low and high temperature) [1,2]. The different stresses can result in cell disturbances and secondary stresses, including membrane damage, protein denaturation, oxidative stress, and osmotic stress at a cellular level [3,4]. To withstand complex environments, plants have evolved complex mechanisms to respond and adapt to these stresses [5]. Among them, heat stress has become one of the main abiotic stresses threatening higher plant productivity and survival throughout the world [6]. The HSP family is one such gene family that is involved in tolerance against heat stress [7,8,9]. However, the molecular mechanisms of the HSP family genes involved in regulating heat stress response are still unclear, and need to be explored.

Heat shock proteins (HSPs) are molecular chaperones induced by heat stress, which are present in all living organisms, from bacteria to protists, plants, fungi, and animals [10,11,12,13]. In plants, the HSP family genes play essential roles in plant growth and development as well as in response to various environmental stresses (heavy metals, salinity, drought, low- and high-temperature stress, and biotic stress) [14,15,16,17]. The HSP gene family has been identified in many plants, such as *Arabidopsis*, soybean (*Glycine max*), foxtail millet (*Setaria italica*), tobacco (*Nicotiana tabacum*), and rice (*Oryza sativa*) [18,19,20,21,22]. The HSP gene family has been divided into five subfamilies based on their molecular weight and domain composition, including the HSP90 subfamily (heat shock protein 90), HSP100 subfamily (the Casein lytic proteinase/heat shock protein 100), HSP20 subfamily (small heat shock proteins), HSP60 (chaperonin proteins), and HSP70 (70-kDa-heat shock proteins) [18].

The roles of *HSP* genes in various stress responses have been reported in many plants [23]. The *Arabidopsis* HSP21, a plastidial small heat shock protein that interacts with the pTAC5 protein, is essential for chloroplast development in *Arabidopsis* under heat stress [24]. The HSP60 protein has been reported to be involved in the folding and aggregation of many proteins that are transported to organelles [25]. The *Arabidopsis* hsp60 mutants displayed severe defects in chloroplast and embryo development and caused cell death [26]. The overexpression of the *SlHSP70-1* gene in transgenic tomato caused internode elongation in tomato [27]. In addition, HSP family genes also play important roles in response to various environmental stresses [28]. The overexpression of the rice *OsMSR3* gene in *Arabidopsis* can enhance tolerance to cadmium and copper stresses [29]. Two *HSP* genes (*CaHSP26* and *CaHSP22.5*) from sweet pepper (*Capsicum annuum*) can protect the PSII system by increasing oxidation resistance and photochemical activity after chilling stress [30]. The overexpression of the *NtHSP* gene in tobacco alleviates NaCl stress, and MeHSP90.9 increases cassava resistance to drought stress by regulating abscisic acid (ABA) and hy0drogen peroxide (H_2_O_2_) [31]. The maize *ZmHSP16.9* gene, encoding a cytosolic class I sHSP, can enhance the heat tolerance in transgenic tobacco [32]. The HSP100/ClpB protein, located in the chloroplast, plays an active role in regulating the heat resistance of tomato (*Solanum lycopersicum*) [33]. In addition, *HSP90* genes in barley play roles in regulating hypersensitive responses to stripe rust [34]. 

*R. delavayi* is an evergreen shrub with large scarlet flowers that make it highly attractive as an ornamental species [35]. However, heat stress causes considerable damages to *R.delavayi*, including leaf scorching, sunburns on leaves and stems, and leaf senescence and abscission [36]. Although the functions of *HSP* genes in some model plants have been well studied, the role of *RdHSP* genes in *R. delavayi* under heat stress remains largely unknown. This study conducted a comprehensive overview of the *RdHSP* gene family in *R. delavayi*, including a phylogenetic analysis, motif/gene structure analysis, chromosome location, gene duplication events, and subcellular localization. We also investigated the expression patterns of the *RdHSP* genes using our RNA-seq data and qRT-PCR to select candidate genes involved in heat stress. Our findings will be useful resources for future studies to unravel the functions of the *RdHSP* genes and will contribute to our understanding of the evolutionary history of the *HSP* genes in different species.

## 2. Materials and Methods

### 2.1. Identification of HSP Family Genes in R. delavayi

The whole genome sequence of six Rhododendron species, including Rhododendron delavayi, Rhododendron williamsianum, Rhododendron simsii, Rhododendron ovatum, Rhododendron henanense, and Rhododendron irroratum, was downloaded from the Ericaceae Genome Resource (TEGR, http://www.tegr.com.cn). The hidden Markov model (HMM) profiles of the HSP20 (PF00011), HSP60 (PF00118), HSP70 (PF00012), HSP90 (PF00183), and HSP100 (PF02861) domains were used as queries to search the genomes of six Rhododendron species. According to the physical localization, the redundant and short sequences (length < 100 aa) were removed. All candidate genes were further screened for the presence of conserved domains using the Pfam and Simple Modular Architecture Research Tool (SMART) databases [37]. Finally, candidate genes with the conserved core domain were used as RdHSP family genes.

### 2.2. Chromosomal Localization, Structure, Cis-Regulatory Elements Analysis 

The locations of *RdHSP* genes were achieved from the *R. delavayi* genome annotation information in the TEGR database. The MapInspect software (http://mapinspect.software.informer.com/) was used to draw the physical location of *RdHSP* genes on chromosomes.

The Gene Structure Display Server (GSDS, version: 2.0) software was used to analyze the intron-exon structures of *RdHSP* genes. The Multiple EM for Motif Elicitation (MEME) website (http://meme-suite.org/tools/meme, accessed on 17 April 2024) was used to identify the conserved motifs of RdHSP family proteins [38].

The upstream 2000 bp sequence of the *RdHSP* genes was manually cut and submitted to the online PlantCARE software (v. 1.0) to predict the cis-regulatory elements. The identified promoter cis-regulatory elements were manually filtered and plotted using GSDS software.

The subcellular localization prediction of the RdHSP family protein was performed using the online WoLF PSORT software (https://wolfpsort.hgc.jp/).

### 2.3. Phylogenetic Analysis

An unrooted phylogenetic tree of the HSP family proteins from six *Rhododendron* species was constructed using the MEGA-X software with the default parameters. The Model Generators were used to estimate the amino acid substitution models for HSPs’ evolution while using the default parameters.

### 2.4. Synteny Analysis

Synteny analyses among six *Rhododendron* species were conducted locally based on the method described [39]. The extracted protein sequences were then aligned pairwise to identify conserved homologous gene pairs using Blastp (v2.7.1) with an E-value threshold of 1E-5. According to the results of homologous gene pairs, MCScanX software was used to identify the syntenic regions [39]. The duplication events of HSPs in six *Rhododendron* species were analyzed using MCScanX software [39].

### 2.5. Expression Patterns of RdHSP Genes in R. delavayi under Heat Stress

The expression patterns of *RdHSPs* in *R. delavayi* leaves under heat stress treatment were investigated using our RNA-seq data. The fragments per kilobase of transcript per million mapped reads (FPKM) was applied to estimate the gene expression. The Cluster 3.0 software was used to perform a hierarchical clustering analysis based on the log_2_ (FPKM), which was visualized using Java TreeView (version:1.2.0).

### 2.6. Real-Time Polymerase Chain Reaction (qRT-PCR) Validation of RNAseq Data

To verify the expression patterns of the 12 candidate *RsHSP* genes in heat-treated *R. delavayi*, RT-qPCR was conducted on the Bio-Rad CFX96 Real-time PCR system using SYBR PrimeScript RT-PCR Kit II (Takara, Dalian, China) and gene specific primers (Table S1). The reference gene Rd18s rRNA was used as a reference for normalization in the qRT-PCR analysis [36]. The 2^−ΔΔCT^ method was applied to calculate the relative expression changes of selected *RdHSP* genes. Relative expression values were obtained from three biological repeats, and measurements were made for three technical repeats.

### 2.7. GO Enrichment Analysis

GOATOOLS software (version:1.2.3) was applied to conduct GO annotations for *RdHSP* genes. The functional enrichment analysis of *RdHSP* genes was performed using Fisher’s exact test. Moreover, Bonferroni correction was applied to minimize false positives, and functions were considered to be significantly enriched when their Bonferroni-corrected P-values (Padjust) were <0.05. The *RdHSP* genes involved in pathways were analyzed using the Kyoto Encyclopedia of Genes and Genomes pathway-mapping database (KEGG; https://www.kegg.jp/kegg/, accessed on 17 April 2024).

### 2.8. Subcellular Localization Analysis of Two Candidate RdHSP Genes

Total RNA extraction from the young leaves of *R. delavayi* was performed, which was reverse transcribed into cDNA using a Plant RNA Kit (TaKaRa Biotechnology Co., Ltd., Dalian, China) and the RT Reagent Kit (TaKaRa Biotechnology Co., Ltd., Dalian, China), respectively. The diluted cDNA (100 ng/μL^−1^) was used as templates for cloning the full-length encoding sequence (CDS) of two candidate *RdHSP*s. The CDS without the stop codon of two *RdHSP20* genes was ligated into a transient expression pCambia 2300 harboring the GFP expression cassette derived by the 35S promoter. Then, the constructed plasmid was transferred to *Agrobacterium* GV3101 through the conventional freezing–thawing method. Next, *Agrobacterium* GV3101 with empty pCambia 2300-GFP or pCambia 2300-RdHSP20s-GFP was injected into 5-week-old *N. benthamiana* leaves. After the infiltrated *N. benthamiana* was cultured in the dark for 24 h and then in low light for 12 h, the GFP signals were detected by an Echo Revolve fluorescence microscope (Revolve FL).

### 2.9. Protein Interaction Prediction

To explore the differential proteins interacting with the RdHSPs involved in regulating *R. delavayi* heat stress response, the RdHSPs associated with heat stress tolerance were identified based on phylogenetic analysis and expression levels, which were submitted to the STRING v10 database (https://string-db.org). Moreover, the construction of the PPI networks was performed based on the Arabidopsis active interaction sources, including experiments, co-expression, and databases. The cytoscape software (v. 3.8.1) was used to visualize the PPI networks. The strong interconnected regions were identified using the ClusterONE software with the parameter sets (minimum density = 0.01, minimum size = 2, and edge weights = combined score). The edges and nodes in the network represent interactions and proteins, respectively. The interactions between the RdHSPs involved in heat stress response and potential differential proteins in *R. delavayi* were screened by STRING.

### 2.10. Statistical Analysis

The experimental design of the article consists of three biological replicates and three technical replicates. Data were statistically analyzed using IBM^®^ SPSS^®^ Statistics 20 (IBM, Armonk, NY, USA). An analysis of variance (ANOVA) and mean separation were performed using a *t*-test or one-way ANOVA with the least significant difference (LSD) at *p* < 0.05.

## 3. Results

### 3.1. Identification and Chromosomal Distribution of the RdHSP Family Genes

**Figure 1:**
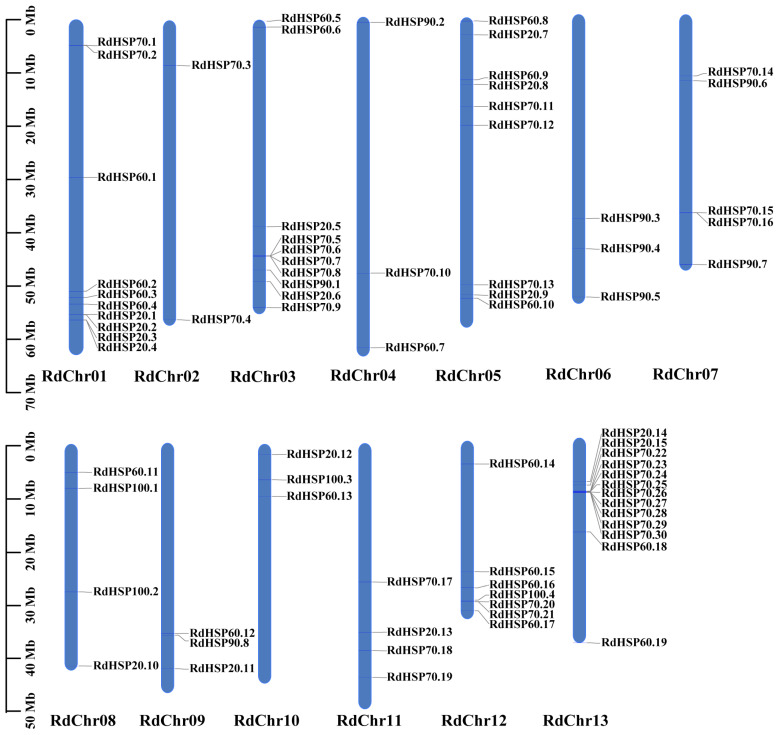
Chromosomal distribution of the RdHSP family genes. The position of each *RdHSP* gene is marked on the right side of each chromosome (Chr). The size of the chromosome is represented by its relative length. Tandemly duplicated gene pairs are indicated with a red bar.

In the present study, a total of 76 *RdHSP* genes were identified in the *R. delavayi* genome (Table S2). The RdHSP proteins ranged in size from 133 aa (RdHSP20.12) to 2558 aa (RdHSP90.7), with an average length of about 632 aa (Table S2). The calculated molecular weight of the predicted RdHSP proteins ranged from 15.01 kDa (RdHSP20.12) to 286.53 kDa (RdHSP90.7), and the isoelectric points of these proteins ranged from 4.7 (RdHSP70.8) to 9.18 (RdHSP20.13) (Table S2). The predicted ranges of the instability index, aliphatic index, and GRAVY of the RdHSP family proteins were 25.32–57.45, 67.78–113.06, and −0.799–0.096, respectively (Table S2). Based on the genome annotation, the identified 76 *RdHSP* genes were unevenly distributed on 13 *R. delavayi* chromosomes. As shown in Figure 1, chromosome 13 contained the largest number of *RdHSP* genes (13), while only two *RdHSP* genes were distributed on chromosome 2. The percentage of *RdHSP* genes per chromosome varied from 0.06% on chromosome 2 to 0.47% on chromosome 13. Interestingly, three tandem duplicated gene pairs, such as *RdHSP70.7* and *RdHSP70.8*, *RdHSP70.15* and *RdHSP70.16*, as well as *RdHSP70.28* and *RdHSP70.29*, were present on chromosomes 03, 07, and 13, respectively.

### 3.2. Phylogenetic Analysis of the HSP Gene Family among Six Rhododendron Species

**Figure 2:**
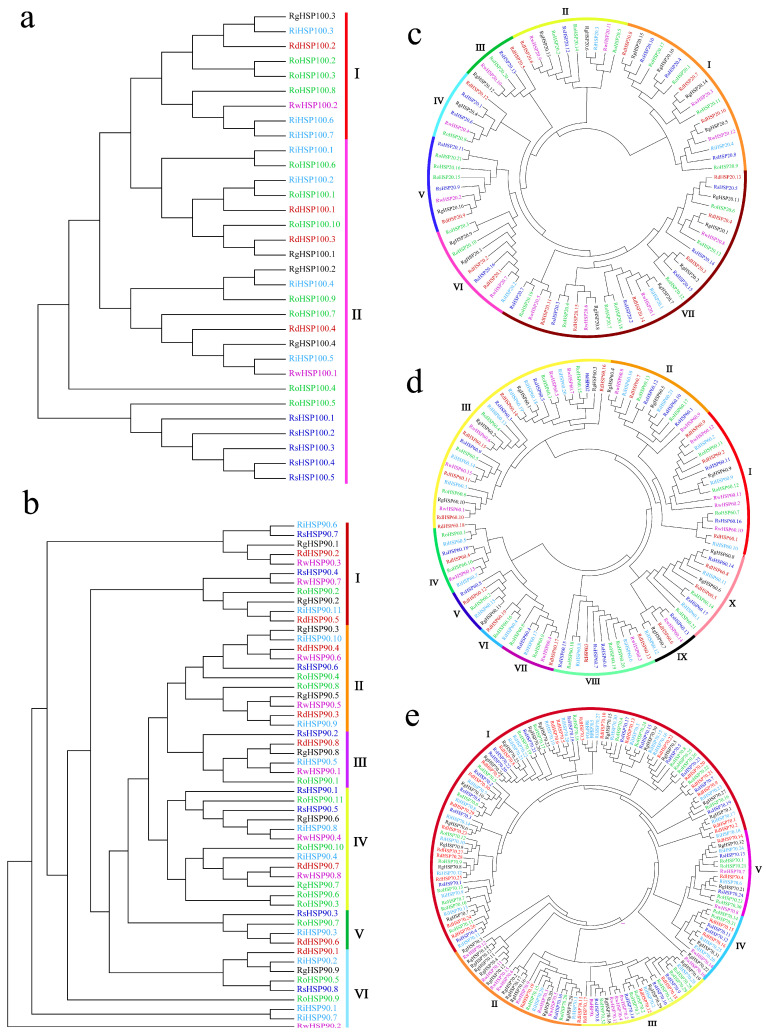
Phylogenetic analysis of HSP subfamily genes. The unrooted phylogenetic trees were constructed using the neighbor-joining method in MEGA 10.0 software with bootstrap test (replicated 1000 times). (**a**) An unrooted phylogenetic tree of the HSP100 subfamily genes; (**b**) an unrooted phylogenetic tree of the HSP90 subfamily genes; (**c**) an unrooted phylogenetic tree of the HSP20 subfamily genes; (**d**) an unrooted phylogenetic tree of the HSP60 subfamily genes; (**e**) an unrooted phylogenetic tree of the HSP70 subfamily genes. Different font colors represent the different Rhododendron species: *R. delavayi* (red), *R. williamsianum* (purple), *R. simsii* (black), *R. ovatum* (blue), *R. henanense* (green), and *R. irroratum* (light blue). Latin numbers (I–X) represent different groups.

**Figure 3:**
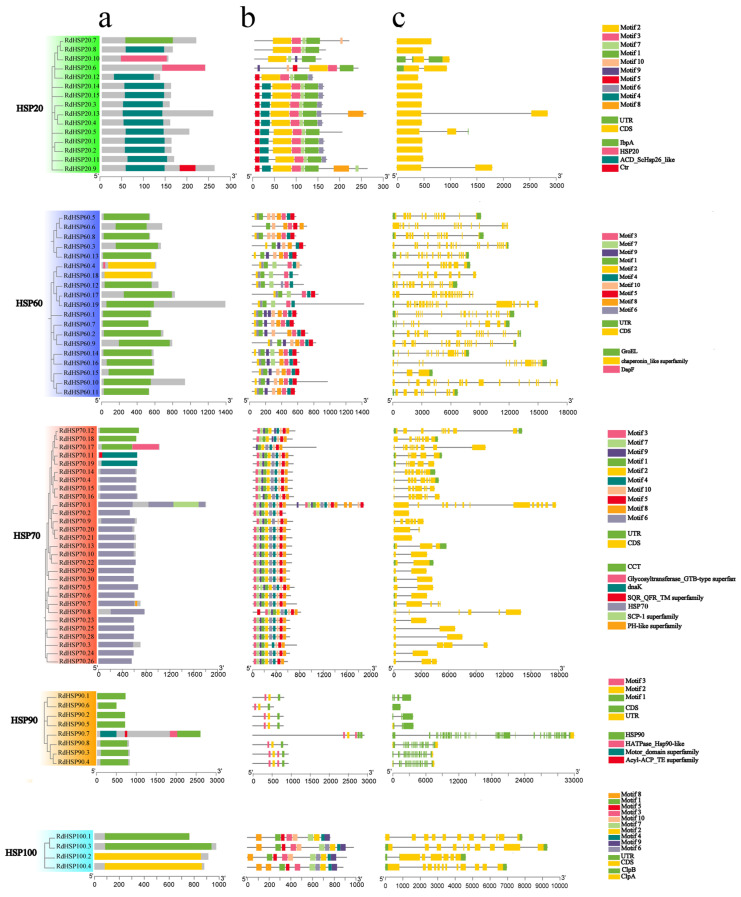
Schematic diagram of the conserved domain, motif composition, and intron–exon distribution of *RdHSP* family genes in *R. delavayi*. (**a**). The conserved domain in classified members of each RdHSP subfamily protein. (**b**). The conserved motifs in each RdHSP protein. Schematic diagram of motif composition in *R. delavayi* RdHSPs was explored using MEME. The relative positions of each motif in RdHSP proteins are shown in different colors. The black lines represent non-conserved sequences. (**c**). Exon–intron distribution of *RdHSP* genes in *R. delavayi*. The exons are represented by orange rectangles. The black lines connecting two exons represent introns.

The unrooted phylogenetic trees of the HSP subfamily genes from six *Rhododendron* species were constructed using the MEGA 10.0 software with the default parameters setting neighbor-joining method (Figure 2). The gene information for *HSP* genes from six *Rhododendron* species was collected and stored in Table S3. Our result showed that the RdHSP family genes of *R. delavayi* were divided into five subfamilies based on domain composition: HSP20, HSP60, HSP70, HSP90, and HSP100 (Figure S1 and Figure 3a). Moreover, each RdHSP subfamily was divided into different groups based on the predicted subcellular localization and phylogenetic tree topology (Figure 2 and Table S2). The RdHSP100 subfamily was classified into two groups: group I (cytoplasm-localized, one gene) and group II (chloroplast-localized, three genes) (Figure 2a and Table S2). The RdHSP90 subfamily was divided into six groups, including group I (cytoplasm-localized, two genes), group II (mitochondrion-localized, two genes), group III (chloroplast-localized, one gene), group IV (endoplasmic reticulum-localized, one gene), group V (mitochondrion-localized, one gene), and group VI (cytoplasm-localized, one gene) (Figure 2b and Table S2). The RdHSP20 subfamily was classified into seven groups: group I (cytoplasm-localized, three genes), group II (chloroplast-localized, one gene), group III (cytoplasm-localized, one gene), group IV (cytoplasm-localized, one gene), group V (plasma membrane-localized, one gene), group VI (cytoplasm-localized, two genes), and group VII (cytoplasm-localized, six genes) (Figure 2c and Table S2). The RdHSP60 subfamily was classified into 10 groups: group I (cytoplasm-localized, 3 genes), group II (chloroplast-localized, 2 genes), group III (cytoplasm-localized, 5 genes), group IV (cytoplasm-localized, 1 gene), group V (endoplasmic reticulum-localized, 1 gene), group VI (cytoplasm-localized, 1 gene), group VII (cytoplasm-localized, 1 gene), group VIII (cytoplasm-localized, 2 genes), group IX (chloroplast-localized, 1 gene), and group X (nucleus-localized, 2 genes) (Figure 2d and Table S2). The RdHSP70 subfamily was classified into 5 groups: group I (chloroplast-localized, 21 genes), group II (mitochondrion-localized, 2 genes), group III (chloroplast-localized, 3 genes), group IV (mitochondrion-localized, 2 genes), and group V (endoplasmic reticulum-localized, 2 genes) (Figure 2e and Table S2). 

### 3.3. Conserved Domain, Protein Motif, and Gene Structure Analyses of the RdHSP Family Genes 

To better analyze the conserved domain, motif composition, and genetic structural diversity of *RdHSP* genes, the distribution of the conserved domain in the RdHSP family proteins was explored (Figure 3a). Our result showed that the RdHSP20, RdHSP60, RdHSP70, RdHSP90, and RdHSP100 subfamily proteins contained the conserved HSP20/α-crystallin domain (PF00011), the GroEL/chaperonin-like (PF00118), CCT/dnaK/HSP70 domain (PF00012), HSP90 (PF00183), and Clp (PF02861), respectively. Furthermore, the motif composition of RdHSP family proteins were investigated using the MEME suite. A total of 10 putative conserved motifs were identified (motifs 1–10, Figure 3b). Motif 1, motif 2, and motif 3 were distributed across all members of the RdHSP20 subfamily. Motif 7 and motif 5 were present in most members of the HSP20 subfamily. All members of the RdHSP60 subfamily contained motif 1, and 94.7% of RdHSP60 subfamily proteins contained motif 2, motif 3, and motif 4. The RdHSP70 subfamily proteins contained motif 1, motif 2, motif 5, motif 8, and motif 9, and the RdHSP90 subfamily proteins contained motif 1, motif 2, and motif 3. The motifs 1–5 and motifs 7–10 were distributed across the RdHSP100 subfamily proteins. As expected, most members in the RdHSP60, RdHSP90, and RdHSP100 subfamilies had similar motif compositions, suggesting functional similarities among the HSP proteins within the same subfamily.

The intron–exon distribution of *RdHSP* family genes was explored and visualized using the Gene Structure Display Server 2.0 (GSDS, Figure 3c). A total of 76 *RdHSP* genes possessed exons varying from 1 to 43. Twelve *RdHSP* genes lacked intron or had only one exon, including *RdHSP20.1*, *RdHSP20.12*, *RdHSP20.14*, *RdHSP20.15*, *RdHSP20.2*, *RdHSP20.3*, *RdHSP20.4*, *RdHSP20.7*, *RdHSP20.8*, *RdHSP70.2*, *RdHSP70.21*, and RdHSP90.6. The majority (62 of 76) of the *RdHSP* genes have 1 to 20 introns, and the *RdHSP90.7* gene contained 43 exons and 42 introns, which was the greatest number of exons in the total *RdHSP* genes. As expected, gene structure analysis revealed that most genes in the same subfamily had similar intron/exon distribution, including the numbers and length of exons. For example, most genes of the RdHSP20 subfamily had one exon and lacked introns. The members of the RdHSP60 subfamily had more than five introns. Thus, our results indicate that *RdHSP* genes in the same subfamily had similar gene structures, which further verify the phylogenetic relationship of these *RdHSP* genes (Figure 2).

### 3.4. Cis-Element Analysis in the RdHSP Gene Promoters in R. delavayi

**Figure 4:**
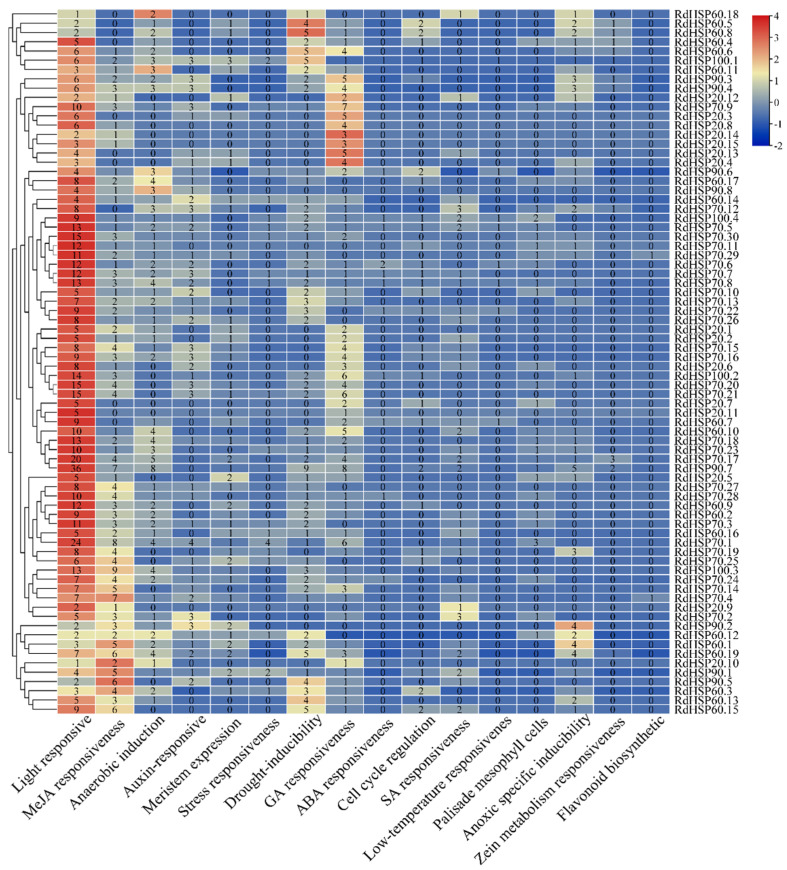
Cis-element analysis in the promoters of the RdHSP family genes. Locations of cis-elements in the 2 kb sequences upstream of *RdHSP* genes. Different kinds of cis-elements are represented with different colored rectangular boxes.

Cis-elements, commonly distributed in 5′ upstream regions of genes, are used as the binding sites of the transcription factors involved in transcriptional regulation. In the present study, the 2000 bp upstream region of the transcription start site (TSS) of *RdHSP* genes was applied to the analysis of cis-regulatory elements using the PlantCARE online database (Figure 4). There was a total of 19 functionally annotated cis-elements in the promoter of most *RdHSP* genes, which were classified into the following four categories: light-responsive elements (Sp1, chs-CMA1a, G-Box, TCCC-motif, Gap-box, TCT-motif, AAC-motif, GT1-motif, and GATA-motif), stress-responsive elements (GC-motif, LTR, TC-rich, MBSI, and MBS), hormone-responsive elements (CGTCA-motif, GACG-motif, TATC-box, GARE-motif, P-box, ABRE, O2-site, and TGA-element), and cis-acting elements related to plant development (AREHD-Zip1, CAT-box, NON-box, and GCN4_motif).

### 3.5. Syntenic Analysis of the RdHSP Gene Family in R. delavayi

**Figure 5:**
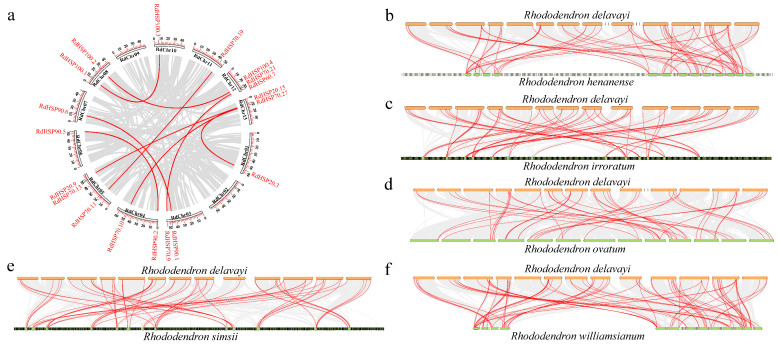
Segmentally duplicated gene pairs of *RdHSP* genes in the *R. delavayi* genome (**a**) and the orthologous relationships of *HSP* genes across six *Rhododendron* species (**b**–**f**). The red lines indicated segmentally duplicated gene pairs (**a**) and orthologous gene pairs (**b**–**f**). The chromosome number is indicated at the top of each chromosome.

To further investigate the potential evolutionary mechanisms of RdHSP family genes, we conducted the collinearity analysis of *RdHSP* genes in the *R. delavayi* genome by the all-vs.-all local BLASTP and MCScan methods (Figure 5). A total of 8 segmental duplication gene pairs with 15 *RdHSP* genes were detected in the *R. delavayi* genome, which only accounted for 19.73% of *RdHSP* family genes (Figure 5a and Table S4). Moreover, all *RdHSP* genes with syntenic regions were distributed on all chromosomes. Subsequently, the ratio of non-synonymous to synonymous substitution (*Ka*/*Ks*) was estimated using the pairwise model by maximum likelihood (PAML v8.0). *Ka*/*Ks* < 1 and *Ka*/*Ks* > 1 represent purifying and positive selection, respectively (Hurst, 2002). In this study, the *Ka*/*Ks* ratios of five *RdHSP* gene pairs were less than one, indicating that purifying selection played an important role in the expansion of these genes, which maintained the similar functions of the *RdHSP* gene family in *R. delavayi*. The *Ka*/*Ks* ratios of three *RdHSP* gene pairs were greater than one, demonstrating that these genes are under positive selection (Table S4). Moreover, *Ks* was usually used to estimate the evolutionary dates of genome or gene duplication events. The WGD/segmental duplicated events in *R. delavayi* occurred from 15.02 (*Ks* = 0.4507) to 90.73 mya (*Ks* = 2.8429).

In addition, to further understand the phylogenetic mechanisms of the *RdHSP* family, comparative syntenic maps were conducted among six *Rhododendron* species (*R. delavayi*, *R. williamsianum*, *R. simsii*, *R. ovatum*, *R. henanense*, and *R. irroratum*) (Figure 5b–e). In the present study, there were 57 orthologous *HSP* gene pairs between *R. delavayi* and *R. henanense* (Table S5), 42 orthologous *HSP* gene pairs between *R. delavayi* and *R. irroratum* (Table S6), 48 orthologous *HSP* gene pairs between *R. delavayi* and *R. ovatum* (Table S7), 46 orthologous *HSP* gene pairs between *R. delavayi* and *R. simsii* (Table S8), and 35 orthologous *HSP* gene pairs between *R. delavayi* and *R. williamsianum* (Table S9). The number of orthologous events of *RdHSP-RgHSP* was much greater than that of *RdHSP-RwHSP*.

**Figure 6:**
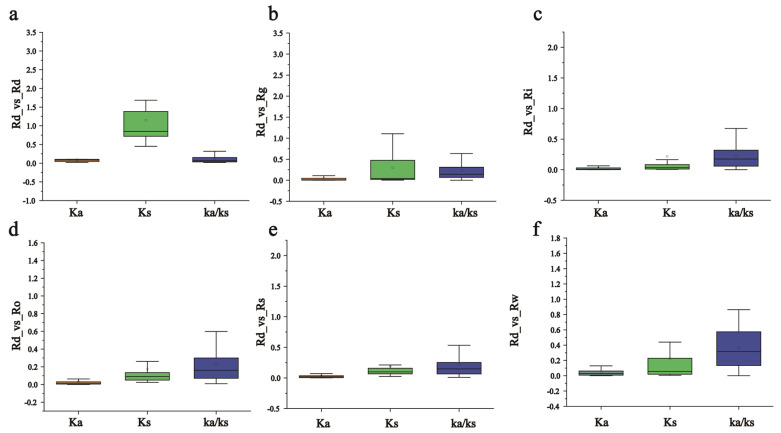
*Ka*, *Ks*, and *Ka*/*Ks* distributions of orthologous HSP gene pairs. (**a**) *Ka*, *Ks*, and *Ka*/*Ks* ratio of segmental duplicated gene pairs; (**b**) orthologous HSP gene pairs between *R. delavayi* and *R. henanense*; (**c**) orthologous HSP gene pairs between *R. delavayi* and *R. irroratum*; (**d**) orthologous HSP gene pairs between *R. delavayi* and *R. ovatum*; (**e**) orthologous HSP gene pairs between *R. delavayi* and *R. simsii*; (**f**) orthologous HSP gene pairs between *R. delavayi* and *R. williamsianum*. The box plots are exhibiting the distributions of *Ka*, *Ks*, and *Ka*/*Ks* values among paralogs and orthologs. The small square and the line in the box represent average and median values of the *Ka*, *Ks*, and *Ka*/*Ks* values, respectively.

The *Ka*, *Ks*, and *Ka*/*Ks* values of the orthologous syntenic gene pairs between peach and strawberry were also computed to analyze the evolutionary selection in the *RdHSP* gene family, and we counted the nonsynonymous (*Ka*) and synonymous substitutions (*Ks*) among the orthologous gene pairs, as well as the *Ka*/*Ks* ratios among the six *Rhododendron* species (Figure 6). The results showed that the *Ka*/*Ks* ratios of the orthologous gene pairs were less than one, suggesting that the orthologous *HSP* genes of the six *Rhododendron* species were subjected to purifying selection during evolution, and similar mechanisms affect the horizontal evolution of HSP family genes across species.

### 3.6. GO and KEGG Enrichment Analysis of RdHSP Genes in R. delavayi

**Figure 7:**
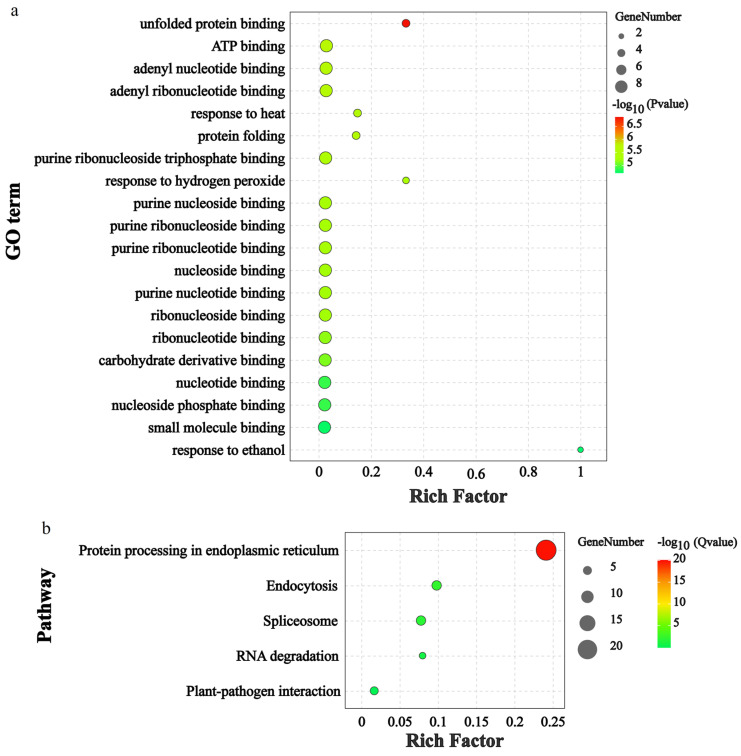
GO and KEGG enrichment analysis. (**a**) GO enrichment analysis of the *RdHSP* genes. (**b**) KEGG pathway enrichment analysis of the *RdHSP* genes. GO, Gene ontology; KEGG, Kyoto Encyclopedia of Genes and Genomes.

Gene ontology helps in the functional analysis of genes by determining their similarity with other genes of known function. In this study, GO enrichment analysis of the *RdHSP* genes was conducted to explore the biological functions of the RdHSP family genes in *R. delavayi* (Figure 7a and Table S10). The top 20 enrichment score GO terms, including 16 molecular functions and 4 biological processes, were enriched in the *RdHSP* genes relative to the complete GO database. In the biological process category, *RdHSP* genes were mainly enriched in response to heat, protein folding, response to hydrogen peroxide, and response to ethanol. In the molecular function category, the *RdHSP* genes were enriched in unfolded protein binding, ATP binding, adenyl nucleotide binding, adenyl ribonucleotide binding, purine ribonucleoside, triphosphate binding, purine nucleoside binding, purine ribonucleoside binding, purine ribonucleotide binding, nucleoside binding, purine nucleotide binding, ribonucleoside binding, ribonucleotide binding, carbohydrate derivative binding, nucleotide binding, nucleoside phosphate binding, and small molecule binding. The GO term enrichment suggested multiple roles of RdHSPs in the cell such as protein processing, growth- and development-related processes, stress responses, and metabolism. Moreover, KEGG enrichment analysis was conducted with *RdHSP* family genes. The twenty-one *RdHSP* genes were significantly enriched in five KEGG pathways, including protein processing in endoplasmic reticulum, lipid and atherosclerosis, endocytosis, spliceosome, RNA degradation, and plant–pathogen interaction (Figure 7b and Table S11), suggesting that these *RdHSP* genes mostly functioned in the regulation of gene expression and immune responses to stress.

### 3.7. Expression Patterns of the RdHSP Family Genes in R. delavayi under High Temperature

**Figure 8:**
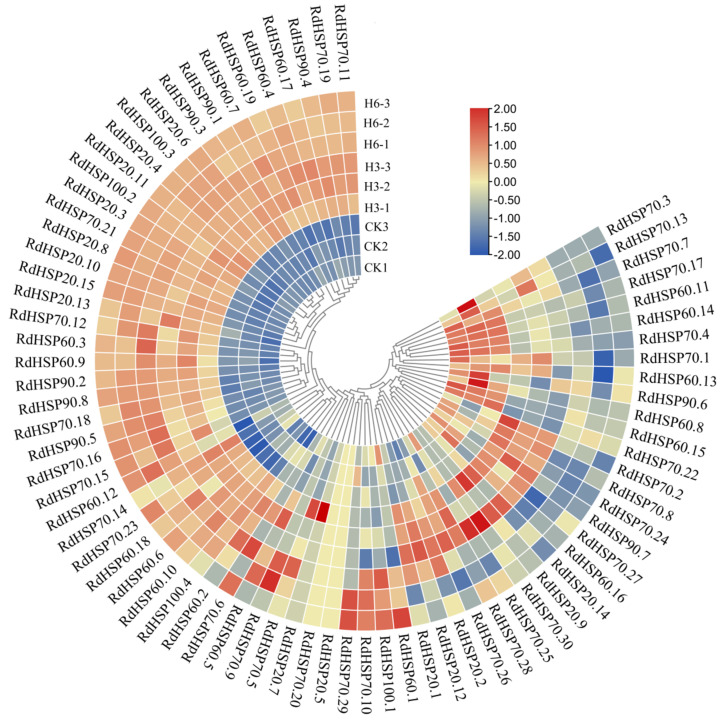
Expression patterns of *RdHSPs* in *R. delavayi* under high-temperature treatments. The scale bars represent the log_2_ transformations of the RPKM values. Light green indicates low expression and red indicates high expression.

To evaluate the expression levels of *RdHSP* genes in *R. delavayi* under high-temperature treatments, the expression patterns of *RdHSP* genes in the leaves of *R. delavayi* treated with a high temperature of 38 °C were evaluated using our RNA-seq data, which were divided into four types (Figure 8). Twenty-one *RdHSP* genes were up-regulated at both 3 d and 6 d under high-temperature treatment, while 10 *RdHSP* genes showed decreased expression at both time points (Figure 8), which may be the long-term response genes in *R. delavayi* under high-temperature treatment. The expression levels of 9 *RdHSP*s (*RdHSP20.4*, *RdHSP20.8*, *RdHSP20.6*, *RdHSP20.3*, *RdHSP20.10*, *RdHSP70.15*, *RdHSP20.15*, *RdHSP70.21*, and *RdHSP70.16*) in *R. delavayi* treated with high temperature were more than 10 times those of the control. Moreover, the expression levels of 28 *RdHSP* genes were increased to a maximum at 3 d, and decreased sharply at 6 d of high-temperature treatment (Figure 8), suggesting that these *RdHSP* genes may be short-term response genes in *R. delavayi* under high-temperature treatment. Both long- and short-term response genes in *R. delavayi* might play important roles during the high-temperature treatment. In addition, there is no significant difference in the expression patterns of 14 *RdHSP* genes between the high-temperature treatment group and the control group.

### 3.8. Validation of RNA-Seq-Based Gene Expression

**Figure 9:**
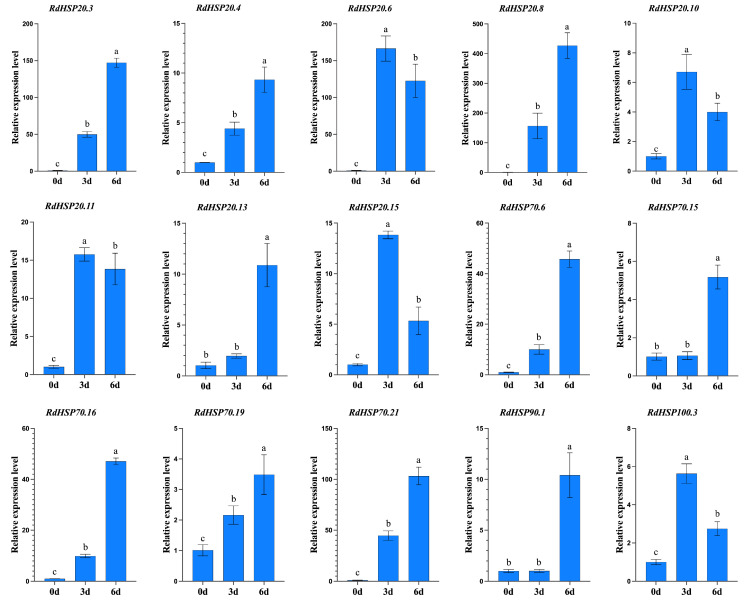
Expression pattern validation of 15 *RdHSPs* in high-temperature treated *R. delavayi* leaves. The expression levels of 15 *RdHSP* genes relative to *18S rRNA* were determined by qRT-PCR. Three technical replicates and three biological replicates were applied for each data point. Data were presented as means ± SD (N = 6). Lowercase letters above bars represent the significant differences between the high-temperature treated *R. delavayi* leaves and control group (*p* < 0.05).

To further confirm the reliability of the RNA-seq results, qRT-PCR was conducted on 15 *RdHSPs* involved in response to heat stress selected at random with high expression levels. Comparative analysis of expression levels was conducted in the leaves of *R. delavayi* treated with high temperature, and the expression trends in the qRT-PCR results were consistent with the RNA-Seq data (Figure 9).

### 3.9. Protein Interaction Prediction

**Figure 10:**
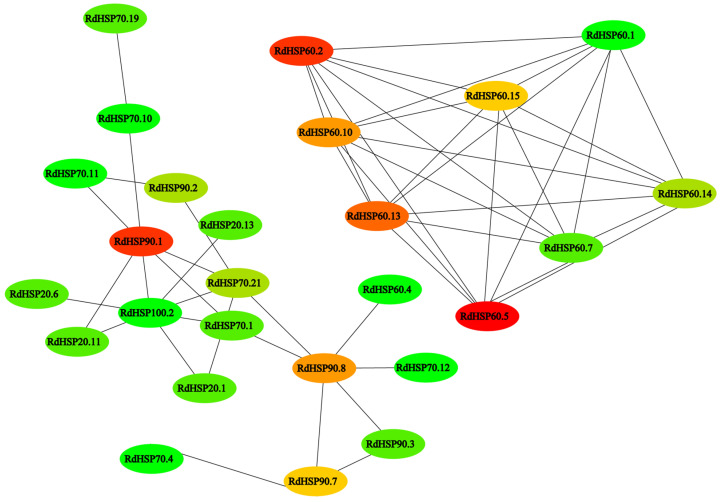
Protein–protein interaction networks among RdHSPs. The black lines represent the interaction strength between proteins.

To further investigate potential protein–protein interactions among RdHSP proteins, we constructed an interaction network of RdHSP family proteins using the STRING database (Figure 10). The results showed that RdHSP60.1 and RdHSP100.20 had the highest number of interactions with other RdHSP proteins, respectively. For example, RdHSP100.2 interacted with seven other RdHSPs such as RdHSP20.11, RdHSP20.1, RdHSP90.1, RdHSP20.13, RdHSP20.6, RdHSP70.1, and RdHSP70.21, respectively. Diverse interactions were also detected in different RdHSP subfamilies, including interacting proteins from the same RdHSP subfamily (e.g., RdHSP60.1 and RdHSP60.10, RdHSP60.1 and RdHSP60.5, RdHSP60.1 and RdHSP60.13, RdHSP60.1 and RdHSP60.15, RdHSP60.1 and RdHSP60.7, RdHSP60.1 and RdHSP60.14, RdHSP60.1 and RdHSP60.2). These findings indicated that the RdHSP family genes may cooperate for regulating various physiological functions and in *R. delavayi*.

### 3.10. Subcellular Location of RdHSP Proteins

**Figure 11:**
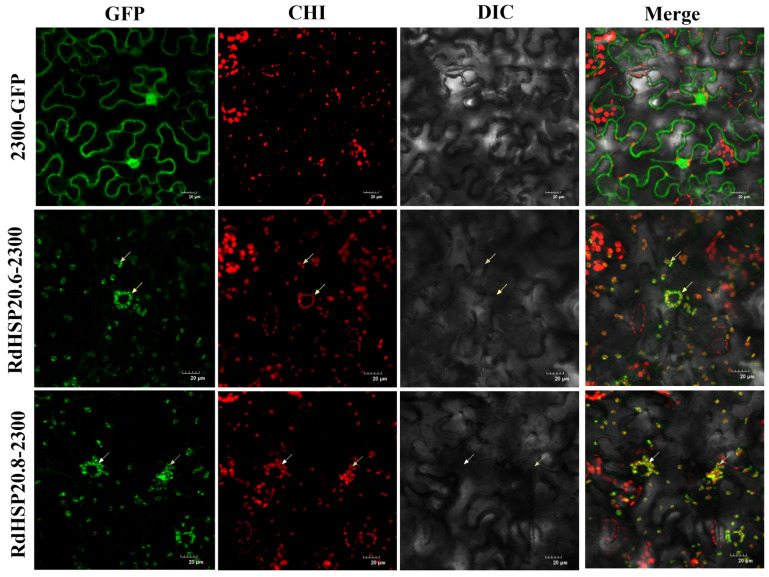
Subcellular localization of two candidate RdHSP proteins (RdHSP20.6 and RdHSP20.8). GFP, CHI, DIC, and Merge represent green fluorescence field (488 nm), chloroplast autofluorescence field, bright field, and superposition field, respectively. The proteins RdHSP20.6 and RdHSP20.8 are localized in the cytoplasm chloroplast indicated by white arrows. Bars of 20 µm.

To verify the prediction results of the subcellular localization of RdHSP proteins, full-length coding sequences of two *RdHSP* genes (*RdHSP20.6* and *RdHSP20.8*) without the stop codon were inserted into the pCAMBIA2300-GFP vector. These two genes were fused with the GFP protein driven by the 35 S promoter, which was transiently expressed in *N. benthamiana* leaves. The subcellular localization results showed that the GFP protein with no gene inserted is expressed in various organelles in *N. benthamiana*, but the GFPs fused with the RdHSP20.6 and RdHSP20.8 proteins are expressed in the chloroplast, indicating that *RdHSP20.6* and *RdHSP20.8* genes are expressed and function in the chloroplast (Figure 11).

## 4. Discussion

The *HSP* family genes play important roles in regulating plant growth and development as well as in response to various environmental stresses [40]. However, a systematic identification of the RdHSP family genes in *R. delavayi* has not been reported. In this study, we identified 76 *RdHSP* genes, including 15 *RdHSP20* genes, 19 *RdHSP60* genes, 30 *RdHSP70* genes, 8 *RdHSP90* genes, and 4 *RdHSP100* genes. The number of HSP family genes varies among different plant species. For example, the number of the RdHSP family genes in *R. delavayi* is slightly higher than that in *Arabidopsis*. This result suggested that no species-specific expansion or gene loss occurred in *R. delavayi*. However, when compared to *R. delavayi*, wheat has a significantly higher number of *HSP* genes [41], suggesting that polyploid organisms may exhibit an elevated number of HSP genes due to gene duplication within each chromosome set. In addition, the number of members in different HSP subfamilies varies greatly among different species (Table S12). The number of genes in the HSP70 subfamily is significantly greater than that in the HSP60 subfamily, suggesting that HSP70 subfamily genes undergo significant expansion.

Gene duplication events such as WGD/segmental duplication, tandem duplication, and dispersed duplication were used as the main important expansion mechanisms for producing new genes and generating genetic novelty in plants [42]. Contrary to expectations, our results showed that 15 (17.44%) and 8 (9.30%) of the RdHSP family genes in *R. delavayi* were obtained from WGD/segmental duplication and tandem duplication, indicating that these two duplication events partially contributed to the expansion of the RdHSP family genes in *R. delavayi*s. Different duplication events contributed differently to the expansion of the HSP gene family in different plants. For example, segmental duplication and tandem duplication events contributed to the expansion of *HSP* genes in cassava and *Triticum aestivum*, respectively [43,44]. In addition, the specific duplication models play different roles in the expansion of the different gene family [45]. For example, segmental duplication and tandem played important roles in expanding the WRKY and AP2/ERF family genes in plant species [46], and transposed duplications contributed to the expansion of the MADS-box and NBS-LRR gene families [45]. Selective pressures played important roles in the evolution of duplicated genes [47], and most duplicated *RdHSP* genes have undergone purification selection. In addition, synteny analysis showed that syntenic *HSP* gene pairs among six *Rhododendron* were detected in this study, and the number of orthologous events of RdHSP-RgHSP was much greater than that of RdHSP-RwHSP, suggesting that *R. henanense* and *R. delavayi* have a closer genetic relationship than *R. williamsianum*. In addition, the *Ka*/*Ks* ratio of segmentally duplicated gene pairs and the orthologous gene pairs was less than one, suggesting that purifying selection played important roles in the expansion of the HSP gene family, and similar mechanisms affect the horizontal evolution of HSP family genes across species. The *Ka*/*Ks* ratio within species was less than the ratio between species, implying that there are significant differences in the horizontal evolution rate of the HSP family genes among different *Rhododendron* species.

Cis-acting regulatory elements (CAREs) in promoters act as key molecular switches for the transcriptional regulation of complex and dynamic gene networks. In this study, analyses of the cis-acting elements in the RdHSP gene promoters were identified and divided into four types: light-, stress-, hormone-, and cis-elements related to plant development. These types of cis-acting regulatory elements were also identified in cassava, dove, and barley [48]. This result further demonstrated that RdHSP family genes played important roles in regulating various physiological processes and in response to complex environmental stresses. In addition, among stress-responsive cis-elements, the cis-element HSE was missing on all promoters of RdHSP family genes, and further revealed the reason why *R. delavayi* exhibits high sensitivity to heat stress.

Numerous studies have revealed that HSP family genes play essential roles in response to high-temperature stress [49]. Our RNA-Seq results revealed that 83.72% (72) of RdHSP family members in *R. delavayi* exhibited the differential expression profiles under high-temperature treatments, indicating that the RdHSP family genes played important roles in regulating high-temperature stress response. Furthermore, 21 *RdHSP* genes were up-regulated at both 3 and 6 d after high- temperature treatment, and the expression level of 28 *RdHSP* genes was increased to a maximum at 3 d, and decreased slightly at 6 d of high-temperature treatment, suggesting that these *RdHSPs* might be associated with the long- and short-term regulation of high-temperature treatment. In addition, the expression levels of nine RdHSP genes in *R. delavayi* treated with high temperature were more than 10 times higher than those of the control, implying that these RdHSPs played key roles in regulating the high-temperature treatment. Functional analysis revealed that the orthologous genes of nine *RdHSP* genes in *Arabidopsis* were involved in response to heat stress [50]. For example, the orthologous genes of *RdHSP20.6*, *RdHSP20.3*, *RdHSP20.10*, *RdHSP70.15*, and *RdHSP70.21 6* in *Arabidopsis* were *HSP21*, *HSP17.6B*, *HSP17.4B*, *BIP2*, and *HSP70-46*, respectively. Previous studies have revealed that *HSP70-4*, *HSP17.4B*, and *HSP21* played important roles in enhancing the heat tolerance, which further confirmed that these genes were involved in enhancing heat tolerance [51,52,53].

## 5. Conclusions

In the present study, the systematic analysis of the *RdHSP* gene family in *R. delavayi* was first performed, and a total of 76 *RdHSP* genes were identified and unevenly distributed on 13 chromosomes. The conserved motifs, gene structure, and evolutionary relationships of the *RdHSP* family genes were also established and analyzed. The exploration of the cis-acting elements in the *RdHSP* gene promoters demonstrated that the *RdHSP* genes played important roles in regulating plant development and in response to various environmental stresses. Expression analysis showed that 83.72% (72) of the *RdHSP* family genes in *R. delavayi* exhibited the differential expression profiles under high-temperature treatments, and the expression levels of 9 *RdHSP* genes (*RdHSP20.4*, *RdHSP20.8*, *RdHSP20.6*, *RdHSP20.3*, *RdHSP20.10*, *RdHSP70.15*, *RdHSP20.15*, *RdHSP70.21*, and *RdHSP70.16*) in high-temperature treated *R. delavayi* were more than 10 times those of the control, suggesting that these genes may be involved in positively regulating heat stress response. The above results could provide a basis for the functional characterization of *HSP* genes, and also provide candidate genes for the future improvement of heat tolerance in *R. delavayi*.

## Data Availability

Data will be made available on request.

## Supplementary Materials

The following supporting information can be downloaded at: https://www.mdpi.com/article/10.3390/plants13131878/s1.
