# Supplementary material for: Genome-Wide Identification and Characterization of RdHSP Genes Related to High Temperature in Rhododendron delavayi
Source: Plants (Basel). 2024 Jul 7;13(13):1878. doi: 10.3390/plants13131878 (PMC11244423; doi:10.3390/plants13131878)
Supplement: Supplementary file 1 [file plants-13-01878-s001.zip › Table S1.pdf]

Table S1. Specific primers used in this study

Gene name	Forward primer (5'to 3')	Reverse primer (5' to 3')
RdHSP20.6	GCCTTCTTTCCGTCCGCAG	TGGTTGCCTTGGTTGCTGA
RdHSP20.8	CCGCCAATGTGCCATCC	CCCGGGCATGTCCACTA
RdHSP20.4	ATCCCAAGCTTCTTCGGCAA	TCGGGGAACGGAATGATGTC
RdHSP70.16	GCATGGTACGGAGTACGACTT	CGCCATCCTTGATCTTCACC
RdHSP20.3	GGAGGAGAAGAACGACAA	CCTCTTTAGGGACGGTAATA
RdHSP90.1	TTTGGGTGGTTGTTTTGCCAG	TCTCCGCAACAAGATACGCC
RdHSP20.11	CTCTCCATCCCACACTCTGA	AAGGTCAGCTTTGAACACATGG
RdHSP70.21	TTGGATGCCGCGGATAAGGGA	GGCACATCACCGCCAACACCAC
RdHSP20.15	ACCACGCTCGTGGACTG	CCCGCTGATCTGCAACA
RdHSP70.15	CTTGGAGTGGTTGGACGATAA	GCCGCTCTTCTCATAAACCT
RdHSP20.10	GCTGTAGATTCGGGCTTGTTC	AATGTCGGCTGGGATACTCG
RdHSP100.3	CGAAGCAACCTCTCTCGTCTCT	CCTGAGAAACAAACCATCGCTAGTC
RdHSP70.6	GGCAATTCGGTGGTTGGATG	TTCAGCATCGCTTCCAATAGC
RdHSP70.19	TCCGTCGCTGGCTCTG	GTGGTACCCAAGTCAATTCCG
RdHSP20.13	CGCGAAGATGGATCAGGTCA	CTCGTAGCGAATCCTCCACC
