# Supplementary material for: Genome-Wide Identification and Characterization of RdHSP Genes Related to High Temperature in Rhododendron delavayi
Source: Plants (Basel). 2024 Jul 7;13(13):1878. doi: 10.3390/plants13131878 (PMC11244423; doi:10.3390/plants13131878)
Supplement: Supplementary file 1 [file plants-13-01878-s001.zip › Table S12.pdf]

Table S12 Numbers of *HSP* genes in ten flowering plants

Species	*Hsp20*	*Hsp60*	*Hsp70*	*Hsp90*	*Hsp100*
T. aestivum	169	95	114	18	84
O. Sativa	39	20	27	8	7
A. thaliana	19	18	18	7	7
L. sativa	32	22	64	7	8
R. delavayi	15	19	30	8	4
R. williamsianum	12	15	23	-	2
R. simsii	16	18	24	8	5
R. ovatum	21	21	32	11	10
R. groenlandicum	16	11	32	8	4
R. irroratum	4	21	30	11	7
