# Supplementary material for: Genome-Wide Identification and Characterization of RdHSP Genes Related to High Temperature in Rhododendron delavayi
Source: Plants (Basel). 2024 Jul 7;13(13):1878. doi: 10.3390/plants13131878 (PMC11244423; doi:10.3390/plants13131878)
Supplement: Supplementary file 1 [file plants-13-01878-s001.zip › Table S3.pdf]

Table S3 The gene name of HSP family members of six *Rhododendron* species

Gene	ID	Gene	ID
Rhdel08G0078100.1	RdHSP100.1	R. irroratum479710.1	RiHSP90.9
Rhdel08G0187700.1	RdHSP100.2	Ro13537.1	RoHSP100.1
Rhdel10G0051600.1	RdHSP100.3	Ro42234.1	RoHSP100.10
Rhdel12G0231700.1	RdHSP100.4	Ro20477.1	RoHSP100.2
Rhdel01G0302300.1	RdHSP20.1	Ro20477.2	RoHSP100.3
Rhdel08G0299600.1	RdHSP20.10	Ro28493.1	RoHSP100.4
Rhdel09G0245500.1	RdHSP20.11	Ro28493.2	RoHSP100.5
Rhdel10G0014800.1	RdHSP20.12	Ro31057.1	RoHSP100.6
Rhdel11G0164200.1	RdHSP20.13	Ro31325.1	RoHSP100.7
Rhdel13G0077600.1	RdHSP20.14	Ro34130.1	RoHSP100.8
Rhdel13G0083100.1	RdHSP20.15	Ro39037.1	RoHSP100.9
Rhdel01G0302500.1	RdHSP20.2	Ro04119.1	RoHSP20.1
Rhdel01G0311000.1	RdHSP20.3	Ro18153.1	RoHSP20.10
Rhdel01G0311200.1	RdHSP20.4	Ro19395.1	RoHSP20.11
Rhdel03G0201700.1	RdHSP20.5	Ro27099.1	RoHSP20.12
Rhdel03G0285200.1	RdHSP20.6	Ro27121.1	RoHSP20.13
Rhdel05G0023800.1	RdHSP20.7	Ro28655.1	RoHSP20.14
Rhdel05G0086500.1	RdHSP20.8	Ro30497.1	RoHSP20.15
Rhdel05G0301500.1	RdHSP20.9	Ro30498.1	RoHSP20.16
Rhdel01G0172500.1	RdHSP60.1	Ro33469.1	RoHSP20.17
Rhdel01G0265700.1	RdHSP60.2	Ro34439.1	RoHSP20.18
Rhdel01G0274700.1	RdHSP60.3	Ro37460.1	RoHSP20.19
Rhdel01G0284500.1	RdHSP60.4	Ro04854.1	RoHSP20.2
Rhdel03G0001700.1	RdHSP60.5	Ro39188.1	RoHSP20.20
Rhdel03G0011300.1	RdHSP60.6	Ro39192.1	RoHSP20.21
Rhdel04G0379300.1	RdHSP60.7	Ro06080.1	RoHSP20.3
Rhdel05G0004300.1	RdHSP60.8	Ro06173.1	RoHSP20.4
Rhdel05G0078400.1	RdHSP60.9	Ro10482.1	RoHSP20.5
Rhdel05G0305100.1	RdHSP60.10	Ro12974.1	RoHSP20.6
Rhdel08G0050200.1	RdHSP60.11	Ro16033.1	RoHSP20.7
Rhdel09G0190500.1	RdHSP60.12	Ro16381.1	RoHSP20.8
Rhdel10G0077700.1	RdHSP60.13	Ro16658.1	RoHSP20.9
Rhdel12G0040100.1	RdHSP60.14	Ro00642.1	RoHSP60.1
Rhdel12G0177400.1	RdHSP60.15	Ro23461.1	RoHSP60.10
Rhdel12G0207500.1	RdHSP60.16	Ro23631.1	RoHSP60.11
Rhdel12G0251500.1	RdHSP60.17	Ro24065.1	RoHSP60.12
Rhdel13G0146800.1	RdHSP60.18	Ro24905.1	RoHSP60.13
Rhdel13G0283500.1	RdHSP60.19	Ro25747.1	RoHSP60.14
Rhdel01G0034900.1	RdHSP70.1	Ro30199.1	RoHSP60.15
Rhdel04G0275700.1	RdHSP70.10	Ro31133.1	RoHSP60.16
Rhdel05G0118700.1	RdHSP70.11	Ro33990.1	RoHSP60.17

Rhdel05G0137500.1	RdHSP70.12	Ro38504.1	RoHSP60.18
Rhdel05G0285700.1	RdHSP70.13	Ro38651.1	RoHSP60.19
Rhdel07G0094500.1	RdHSP70.14	Ro02323.1	RoHSP60.2
Rhdel07G0248700.1	RdHSP70.15	Ro38651.2	RoHSP60.20
Rhdel07G0248800.1	RdHSP70.16	Ro40324.1	RoHSP60.21
Rhdel11G0099300.1	RdHSP70.17	Ro06566.1	RoHSP60.3
Rhdel11G0188600.1	RdHSP70.18	Ro10283.1	RoHSP60.4
Rhdel11G0223000.1	RdHSP70.19	Ro14722.1	RoHSP60.5
Rhdel01G0035400.1	RdHSP70.2	Ro15461.1	RoHSP60.6
Rhdel12G0234800.1	RdHSP70.20	Ro18634.1	RoHSP60.7
Rhdel12G0235100.1	RdHSP70.21	Ro20052.1	RoHSP60.8
Rhdel13G0092800.1	RdHSP70.22	Ro20052.2	RoHSP60.9
Rhdel13G0093100.1	RdHSP70.23	Ro02944.1	RoHSP70.1
Rhdel13G0093200.1	RdHSP70.24	Ro06082.1	RoHSP70.10
Rhdel13G0093500.1	RdHSP70.25	Ro06083.1	RoHSP70.11
Rhdel13G0094200.1	RdHSP70.26	Ro06107.1	RoHSP70.12
Rhdel13G0094300.1	RdHSP70.27	Ro06146.1	RoHSP70.13
Rhdel13G0094500.1	RdHSP70.28	Ro06193.1	RoHSP70.14
Rhdel13G0094600.1	RdHSP70.29	Ro07030.1	RoHSP70.15
Rhdel02G0062000.1	RdHSP70.3	Ro07918.1	RoHSP70.16
Rhdel13G0094800.1	RdHSP70.30	Ro11104.1	RoHSP70.17
Rhdel02G0336700.1	RdHSP70.4	Ro11108.1	RoHSP70.18
Rhdel03G0244200.1	RdHSP70.5	Ro11122.1	RoHSP70.19
Rhdel03G0244600.1	RdHSP70.6	Ro03950.1	RoHSP70.2
Rhdel03G0244700.1	RdHSP70.7	Ro16185.1	RoHSP70.20
Rhdel03G0244800.1	RdHSP70.8	Ro18443.1	RoHSP70.21
Rhdel03G0332900.1	RdHSP70.9	Ro19694.1	RoHSP70.22
Rhdel03G0265600.1	RdHSP90.1	Ro19762.1	RoHSP70.23
Rhdel04G0008000.1	RdHSP90.2	Ro23705.1	RoHSP70.24
Rhdel06G0205800.1	RdHSP90.3	Ro23998.1	RoHSP70.25
Rhdel06G0249500.1	RdHSP90.4	Ro23998.2	RoHSP70.26
Rhdel06G0325900.1	RdHSP90.5	Ro27243.1	RoHSP70.27
Rhdel07G0103300.1	RdHSP90.6	Ro27245.1	RoHSP70.28
Rhdel07G0321100.1	RdHSP90.7	Ro27245.2	RoHSP70.29
Rhdel09G0194000.1	RdHSP90.8	Ro03950.2	RoHSP70.3
cds-Rhe021743.1	RgHSP100.1	Ro34992.1	RoHSP70.30
cds-Rhe023790.1	RgHSP100.2	Ro36601.1	RoHSP70.31
cds-Rhe024416.1	RgHSP100.3	Ro40407.1	RoHSP70.32
cds-Rhe028343.1	RgHSP100.4	Ro04918.1	RoHSP70.4
cds-Rhe000585.1	RgHSP20.1	Ro06068.1	RoHSP70.5
cds-Rhe029262.1	RgHSP20.10	Ro06070.1	RoHSP70.6
cds-Rhe008935.1	RgHSP20.11	Ro06073.1	RoHSP70.7
cds-Rhe013464.1	RgHSP20.12	Ro06079.1	RoHSP70.8
cds-Rhe014127.1	RgHSP20.13	Ro06081.1	RoHSP70.9

cds-Rhe014784.1	RgHSP20.14	Ro02336.1	RoHSP90.1
cds-Rhe015231.1	RgHSP20.15	Ro30261.1	RoHSP90.10
cds-Rhe016524.1	RgHSP20.16	Ro30271.1	RoHSP90.11
cds-Rhe000586.1	RgHSP20.2	Ro03577.1	RoHSP90.2
cds-Rhe000645.1	RgHSP20.3	Ro05386.1	RoHSP90.3
cds-Rhe021434.1	RgHSP20.4	Ro07264.1	RoHSP90.4
cds-Rhe023373.1	RgHSP20.5	Ro08903.1	RoHSP90.5
cds-Rhe025616.1	RgHSP20.6	Ro09084.1	RoHSP90.6
cds-Rhe026390.1	RgHSP20.7	Ro18590.1	RoHSP90.7
cds-Rhe026445.1	RgHSP20.8	Ro19745.1	RoHSP90.8
cds-Rhe026518.1	RgHSP20.9	Ro26490.1	RoHSP90.9
cds-Rhe028511.1	RgHSP60.1	cds-KAE9453676.1	RwHSP100.1
cds-Rhe016555.1	RgHSP60.10	cds-KAE9453875.1	RwHSP100.2
cds-Rhe020511.1	RgHSP60.11	cds-KAE9444829.1	RwHSP20.1
cds-Rhe028712.1	RgHSP60.2	cds-KAE9465246.1	RwHSP20.10
cds-Rhe029661.1	RgHSP60.3	cds-KAE9466269.1	RwHSP20.11
cds-Rhe002720.1	RgHSP60.4	cds-KAE9467975.1	RwHSP20.12
cds-Rhe007031.1	RgHSP60.5	cds-KAE9447736.1	RwHSP20.2
cds-Rhe012171.1	RgHSP60.6	cds-KAE9448813.1	RwHSP20.3
cds-Rhe012227.1	RgHSP60.7	cds-KAE9449066.1	RwHSP20.4
cds-Rhe014683.1	RgHSP60.8	cds-KAE9451407.1	RwHSP20.5
cds-Rhe015163.1	RgHSP60.9	cds-KAE9461056.1	RwHSP20.6
cds-Rhe002346.1	RgHSP70.1	cds-KAE9463534.1	RwHSP20.7
cds-Rhe026530.1	RgHSP70.10	cds-KAE9463568.1	RwHSP20.8
cds-Rhe027154.1	RgHSP70.11	cds-KAE9464694.1	RwHSP20.9
cds-Rhe028213.1	RgHSP70.12	cds-KAE9447701.1	RwHSP60.1
cds-Rhe029827.1	RgHSP70.13	cds-KAE9463059.1	RwHSP60.10
cds-Rhe029882.1	RgHSP70.14	cds-KAE9463310.1	RwHSP60.11
cds-Rhe003381.1	RgHSP70.15	cds-KAE9463313.1	RwHSP60.12
cds-Rhe005305.1	RgHSP70.16	cds-KAE9463448.1	RwHSP60.13
cds-Rhe005310.1	RgHSP70.17	cds-KAE9466003.1	RwHSP60.14
cds-Rhe008432.1	RgHSP70.18	cds-KAE9466404.1	RwHSP60.15
cds-Rhe009066.1	RgHSP70.19	cds-KAE9448217.1	RwHSP60.2
cds-Rhe021462.1	RgHSP70.2	cds-KAE9449438.1	RwHSP60.3
cds-Rhe009275.1	RgHSP70.20	cds-KAE9453476.1	RwHSP60.4
cds-Rhe009871.1	RgHSP70.21	cds-KAE9453799.1	RwHSP60.5
cds-Rhe011959.1	RgHSP70.22	cds-KAE9453926.1	RwHSP60.6
cds-Rhe013773.1	RgHSP70.23	cds-KAE9454682.1	RwHSP60.7
cds-Rhe013774.1	RgHSP70.24	cds-KAE9455144.1	RwHSP60.8
cds-Rhe013776.1	RgHSP70.25	cds-KAE9457200.1	RwHSP60.9
cds-Rhe013777.1	RgHSP70.26	cds-KAE9446375.1	RwHSP70.1
cds-Rhe014543.1	RgHSP70.27	cds-KAE9446844.1	RwHSP70.2
cds-Rhe015476.1	RgHSP70.28	cds-KAE9447018.1	RwHSP70.3
cds-Rhe015614.1	RgHSP70.29	cds-KAE9448315.1	RwHSP70.4

cds-Rhe022679.1	RgHSP70.3	cds-KAE9448397.1	RwHSP70.5
cds-Rhe016430.1	RgHSP70.30	cds-KAE9449043.1	RwHSP70.6
cds-Rhe017390.1	RgHSP70.31	cds-KAE9451098.1	RwHSP70.7
cds-Rhe018502.1	RgHSP70.32	cds-KAE9451121.1	RwHSP70.8
cds-Rhe023614.1	RgHSP70.4	cds-KAE9451878.1	RwHSP70.9
cds-Rhe026522.1	RgHSP70.5	cds-KAE9452527.1	RwHSP70.10
cds-Rhe026525.1	RgHSP70.6	cds-KAE9453176.1	RwHSP70.11
cds-Rhe026526.1	RgHSP70.7	cds-KAE9453464.1	RwHSP70.12
cds-Rhe026528.1	RgHSP70.8	cds-KAE9453577.1	RwHSP70.13
cds-Rhe026529.1	RgHSP70.9	cds-KAE9454778.1	RwHSP70.14
cds-Rhe004955.1	RgHSP90.1	cds-KAE9456571.1	RwHSP70.15
cds-Rhe005151.1	RgHSP90.2	cds-KAE9456949.1	RwHSP70.16
cds-Rhe005770.1	RgHSP90.3	cds-KAE9457593.1	RwHSP70.17
cds-Rhe006092.1	RgHSP90.5	cds-KAE9457660.1	RwHSP70.18
cds-Rhe007343.1	RgHSP90.6	cds-KAE9457856.1	RwHSP70.19
cds-Rhe016923.1	RgHSP90.7	cds-KAE9458373.1	RwHSP70.20
cds-Rhe020532.1	RgHSP90.8	cds-KAE9458643.1	RwHSP70.21
cds-Rhe030430.1	RgHSP90.9	cds-KAE9459003.1	RwHSP70.22
Rhododendron_irroratum248820.1	RiHSP100.1	cds-KAE9464825.1	RwHSP70.23
Rhododendron_irroratum280760.1	RiHSP100.2	RHSIM_RhsimUnG0004900	RsHSP20.1
Rhododendron_irroratum292310.1	RiHSP100.3	RHSIM_Rhsim05G0073400	RsHSP20.10
Rhododendron_irroratum299820.1	RiHSP100.4	RHSIM_Rhsim03G0138600	RsHSP20.11
Rhododendron_irroratum428170.1	RiHSP100.5	RHSIM_Rhsim03G0214500	RsHSP20.12
Rhododendron_irroratum432340.1	RiHSP100.6	RHSIM_Rhsim03G0138500	RsHSP20.13
Rhododendron_irroratum432390.1	RiHSP100.7	RHSIM_Rhsim01G0233900	RsHSP20.14
Rhododendron_irroratum103420.1	RiHSP20.1	RHSIM_Rhsim01G0233700	RsHSP20.15
Rhododendron_irroratum153840.1	RiHSP20.2	RHSIM_Rhsim01G0227000	RsHSP20.16
Rhododendron_irroratum274410.1	RiHSP20.3	RHSIM_Rhsim13G0068500	RsHSP20.2
Rhododendron_irroratum304930.1	RiHSP20.4	RHSIM_Rhsim13G0075700	RsHSP20.3
Rhododendron_irroratum002260.1	RiHSP60.1	RHSIM_Rhsim12G0087000	RsHSP20.4
Rhododendron_irroratum168470.1	RiHSP60.10	RHSIM_Rhsim11G0107000	RsHSP20.5
Rhododendron_irroratum189000.1	RiHSP60.11	RHSIM_Rhsim10G0013000	RsHSP20.6
Rhododendron_irroratum189830.1	RiHSP60.12	RHSIM_Rhsim09G0170000	RsHSP20.7
Rhododendron_irroratum227980.1	RiHSP60.13	RHSIM_Rhsim08G0242400	RsHSP20.8
Rhododendron_irroratum277780.1	RiHSP60.14	RHSIM_Rhsim05G0200800	RsHSP20.9
Rhododendron_irroratum329530.1	RiHSP60.15	RHSIM_RhsimUnG0194000	RsHSP60.1
Rhododendron_irroratum379870.1	RiHSP60.16	RHSIM_Rhsim06G0057200	RsHSP60.10
Rhododendron_irroratum426100.1	RiHSP60.17	RHSIM_Rhsim05G0064500	RsHSP60.11
Rhododendron_irroratum430590.1	RiHSP60.18	RHSIM_Rhsim04G0232000	RsHSP60.12
Rhododendron_irroratum430900.1	RiHSP60.19	RHSIM_Rhsim03G0008300	RsHSP60.13
Rhododendron_irroratum011310.1	RiHSP60.2	RHSIM_Rhsim03G0001500	RsHSP60.14
Rhododendron_irroratum448490.1	RiHSP60.20	RHSIM_Rhsim01G0205900	RsHSP60.15
Rhododendron_irroratum464350.1	RiHSP60.21	RHSIM_Rhsim01G0136000	RsHSP60.16
Rhododendron_irroratum034730.1	RiHSP60.3	RHSIM_Rhsim01G0190500	RsHSP60.17

Rhododendron_irroratum079640.1	RiHSP60.4	RHSIM_Rhsim01G0213500	RsHSP60.18
Rhododendron_irroratum096080.1	RiHSP60.5	RHSIM_Rhsim12G0163900	RsHSP60.2
Rhododendron_irroratum136080.1	RiHSP60.6	RHSIM_Rhsim12G0140600	RsHSP60.3
Rhododendron_irroratum155300.1	RiHSP60.7	RHSIM_Rhsim12G0202100	RsHSP60.4
Rhododendron_irroratum156200.1	RiHSP60.8	RHSIM_Rhsim12G0038400	RsHSP60.5
Rhododendron_irroratum157060.1	RiHSP60.9	RHSIM_Rhsim10G0063000	RsHSP60.6
Rhododendron_irroratum015960.1	RiHSP70.1	RHSIM_Rhsim10G0066200	RsHSP60.7
Rhododendron_irroratum101770.1	RiHSP70.10	RHSIM_Rhsim09G0117900	RsHSP60.8
Rhododendron_irroratum101800.1	RiHSP70.11	RHSIM_Rhsim08G0045300	RsHSP60.9
Rhododendron_irroratum101820.1	RiHSP70.12	RHSIM_RhsimUnG0065400	RsHSP90.1
Rhododendron_irroratum101830.1	RiHSP70.13	RHSIM_Rhsim09G0120100	RsHSP90.2
Rhododendron_irroratum101840.1	RiHSP70.14	RHSIM_Rhsim07G0087900	RsHSP90.3
Rhododendron_irroratum101860.1	RiHSP70.15	RHSIM_Rhsim06G0232600	RsHSP90.4
Rhododendron_irroratum101960.1	RiHSP70.16	RHSIM_Rhsim06G0020200	RsHSP90.5
Rhododendron_irroratum184810.1	RiHSP70.17	RHSIM_Rhsim06G0174600	RsHSP90.6
Rhododendron_irroratum184820.1	RiHSP70.18	RHSIM_Rhsim04G0010300	RsHSP90.7
Rhododendron_irroratum216010.1	RiHSP70.19	RHSIM_Rhsim03G0191300	RsHSP90.8
Rhododendron_irroratum018020.1	RiHSP70.2	RHSIM_Rhsim12G0182500	RsHSP100.1
Rhododendron_irroratum216020.1	RiHSP70.20	RHSIM_Rhsim10G0045100	RsHSP100.1
Rhododendron_irroratum216110.1	RiHSP70.21	RHSIM_Rhsim08G0200500	RsHSP100.1
Rhododendron_irroratum216150.1	RiHSP70.22	RHSIM_Rhsim08G0147100	RsHSP100.1
Rhododendron_irroratum225310.1	RiHSP70.23	RHSIM_Rhsim08G0196000	RsHSP100.1
Rhododendron_irroratum238290.1	RiHSP70.24	RHSIM_RhsimUnG0190400	RsHSP70.1
Rhododendron_irroratum253790.1	RiHSP70.25	RHSIM_Rhsim13G0085000	RsHSP70.2
Rhododendron_irroratum253800.1	RiHSP70.26	RHSIM_Rhsim13G0084400	RsHSP70.3
Rhododendron_irroratum262860.1	RiHSP70.27	RHSIM_Rhsim13G0084600	RsHSP70.4
Rhododendron_irroratum366770.1	RiHSP70.28	RHSIM_Rhsim13G0084100	RsHSP70.5
Rhododendron_irroratum369750.1	RiHSP70.29	RHSIM_Rhsim13G0084900	RsHSP70.6
Rhododendron_irroratum032940.1	RiHSP70.3	RHSIM_Rhsim12G0185700	RsHSP70.7
Rhododendron_irroratum390550.1	RiHSP70.30	RHSIM_Rhsim11G0051900	RsHSP70.8
Rhododendron_irroratum047460.1	RiHSP70.4	RHSIM_Rhsim11G0122200	RsHSP70.9
Rhododendron_irroratum047970.1	RiHSP70.5	RHSIM_Rhsim11G0122300	RsHSP70.10
Rhododendron_irroratum078110.1	RiHSP70.6	RHSIM_Rhsim07G0187100	RsHSP70.11
Rhododendron_irroratum101740.1	RiHSP70.7	RHSIM_Rhsim07G0187200	RsHSP70.12
Rhododendron_irroratum101750.1	RiHSP70.8	RHSIM_Rhsim07G0079900	RsHSP70.13
Rhododendron_irroratum101760.1	RiHSP70.9	RHSIM_Rhsim05G0113400	RsHSP70.14
Rhododendron_irroratum011270.1	RiHSP90.1	RHSIM_Rhsim05G0190800	RsHSP70.15
Rhododendron_irroratum484800.1	RiHSP90.10	RHSIM_Rhsim05G0096400	RsHSP70.16
Rhododendron_irroratum492500.1	RiHSP90.11	RHSIM_Rhsim04G0156300	RsHSP70.17
Rhododendron_irroratum218390.1	RiHSP90.2	RHSIM_Rhsim03G0170200	RsHSP70.18
Rhododendron_irroratum239170.1	RiHSP90.3	RHSIM_Rhsim03G0258400	RsHSP70.19
Rhododendron_irroratum261230.1	RiHSP90.4	RHSIM_Rhsim03G0170400	RsHSP70.20
Rhododendron_irroratum329730.1	RiHSP90.5	RHSIM_Rhsim03G0170600	RsHSP70.21
Rhododendron_irroratum421830.1	RiHSP90.6	RHSIM_Rhsim03G0170500	RsHSP70.22

Rhododendron_irroratum424140.1	RiHSP90.7	RHSIM_Rhsim03G0131600	RsHSP70.23
Rhododendron_irroratum458980.1	RiHSP90.8	RHSIM_Rhsim02G0248700	RsHSP70.24
