# Supplementary material for: Genome-Wide Identification and Characterization of RdHSP Genes Related to High Temperature in Rhododendron delavayi
Source: Plants (Basel). 2024 Jul 7;13(13):1878. doi: 10.3390/plants13131878 (PMC11244423; doi:10.3390/plants13131878)
Supplement: Supplementary file 1 [file plants-13-01878-s001.zip › Table S4.pdf]

**Table S4 Segmentally duplicated RdHSP gene pairs**

| Seq_1 | Seq_2 | Ka | Ks | Ka_Ks |
|---|---|---|---|---|
| RdHSP20. 3 | RdHSP20. 15 | 0. 065636716 | 0. 450773919 | 0. 145608947 |
| RdHSP20. 9 | RdHSP20. 15 | 0. 258853112 | 0. 811783624 | 0. 318869591 |
| RdHSP90. 1 | RdHSP90. 6 | 0. 093132419 | 2. 842882384 | 0. 032759856 |
| RdHSP90. 2 | RdHSP90. 5 | 0. 028250193 | 0. 791005293 | 0. 03571429 |
| RdHSP100. 3 | RdHSP100. 1 | 0. 103505872 | 0. 646891313 | 0. 160005043 |
| RdHSP70. 13 | RdHSP70. 10 | 0. 019824203 | 1. 078839615 | 0. 018375486 |
| RdHSP70. 19 | RdHSP70. 11 | 0. 063227214 | 0. 89154815 | 0. 070918451 |
| RdHSP70. 21 | RdHSP70. 9 | 0. 104630875 | 1. 685022129 | 0. 062094659 |
