# Supplementary material for: Genome-Wide Identification and Characterization of RdHSP Genes Related to High Temperature in Rhododendron delavayi
Source: Plants (Basel). 2024 Jul 7;13(13):1878. doi: 10.3390/plants13131878 (PMC11244423; doi:10.3390/plants13131878)
Supplement: Supplementary file 1 [file plants-13-01878-s001.zip › Table S5.pdf]

Table S5 The orthologous relationships of the HSP genes between *R. delavayi and R. henanense*

Seq_1	Seq_2	Ka	Ks	Ka_Ks
RgHSP20.1	RdHSP20.3	0.020985281	0.219222003	0.095726164
RgHSP20.3	RdHSP20.1	0.013498677	0.039777341	0.339355941
RgHSP20.1	RdHSP20.11	0.113666676	1.351382691	0.084111389
RgHSP20.1	RdHSP20.15	0.054608743	0.427623798	0.127702769
RgHSP60.4	RdHSP60.7	0.00081928	0.011202075	0.073136492
RgHSP70.15	RdHSP70.10	0.002977671	0.053498609	0.055658857
RgHSP90.1	RdHSP90.2	0.002418138	0.025416371	0.095140966
RgHSP70.15	RdHSP70.13	0.022496231	1.105381167	0.02035156
RgHSP90.1	RdHSP90.5	0.028870388	0.812054205	0.035552292
RgHSP90.2	RdHSP90.2	0.029505956	0.771073878	0.038266056
RgHSP90.5	RdHSP90.3	0.005450853	0.021097763	0.258361653
RgHSP90.2	RdHSP90.5	0.002407224	0.042497598	0.056643759
RgHSP70.20	RdHSP70.11	0.064554748	0.921900362	0.070023563
RgHSP70.20	RdHSP70.19	0.003229284	0.010309441	0.313235595
RgHSP70.21	RdHSP70.4	0.00386495	0.034285154	0.112729559
RgHSP70.21	RdHSP70.14	0.038919166	0.783600516	0.049667101
RgHSP20.12	RdHSP20.5	0.132079427	0.183401056	0.720167209
RgHSP70.23	RdHSP70.5	0.02971712	0.071866743	0.413503089
RgHSP20.13	RdHSP20.6	0.007350748	0.031092535	0.236415201
RgHSP70.27	RdHSP70.9	0.018404753	0.060573299	0.30384267
RgHSP60.6	RdHSP60.5	0	0.007915641	0
RgHSP60.7	RdHSP60.6	0.134763743	0.197050729	0.683903805
RgHSP70.30	RdHSP70.10	0.019824203	1.059760685	0.018706301
RgHSP60.8	RdHSP60.8	0	0.021296906	0
RgHSP20.14	RdHSP20.7	0.011133808	0.017524162	0.635340388
RgHSP60.9	RdHSP60.9	0.002359729	0.035781913	0.065947543
RgHSP20.15	RdHSP20.8	0.008178182	0.12007147	0.06811095
RgHSP70.28	RdHSP70.11	0.001290323	0.004155135	0.310536917
RgHSP70.29	RdHSP70.12	0.001864513	0.015666366	0.119013758
RgHSP70.30	RdHSP70.13	0.001314493	0.044250317	0.029705835
RgHSP20.16	RdHSP20.9	0.024716195	0.029463691	0.83886964
RgHSP70.28	RdHSP70.19	0.062634538	0.904705726	0.069231946
RgHSP70.32	RdHSP70.14	0.002570421	0.011316699	0.227135251
RgHSP70.31	RdHSP70.15	0.005195107	0.051292074	0.101284793
RgHSP60.11	RdHSP60.12	0.015173248	0.020662732	0.734329232
RgHSP90.8	RdHSP90.8	0.008311629	0.010421357	0.797557277
RgHSP100.1	RdHSP100.1	0.110047455	0.644316534	0.170797192
RgHSP20.4	RdHSP20.12	0.094471848	0.150266652	0.628694699
RgHSP100.1	RdHSP100.3	0.003134567	0.014631042	0.214240849
RgHSP20.5	RdHSP20.10	0.005689928	0.03981034	0.142925877

RgHSP100.3	RdHSP100.2	0.00479388	0.009384898	0.5108079
RgHSP100.2	RdHSP100.4	0.16628301	0.662041018	0.251167232
RgHSP20.8	RdHSP20.3	0.068503314	0.474416307	0.144394939
RgHSP20.9	RdHSP20.1	0.072038396	0.601385626	0.11978736
RgHSP70.9	RdHSP70.13	0.105175899	2.580804778	0.04075314
RgHSP20.8	RdHSP20.9	0.262031731	0.823959217	0.318015414
RgHSP20.8	RdHSP20.11	0.130298876	1.162766944	0.112059323
RgHSP20.7	RdHSP20.14	0.016319774	0.071726974	0.227526307
RgHSP20.8	RdHSP20.15	0.010835403	0.040210634	0.269466103
RgHSP70.5	RdHSP70.22	0.000662691	0.013657434	0.048522335
RgHSP70.6	RdHSP70.23	0.025158849	0.046516791	0.540855211
RgHSP70.7	RdHSP70.24	0.00771711	0.044320586	0.174120215
RgHSP70.8	RdHSP70.25	0.046493206	0.149682453	0.31061227
RgHSP100.4	RdHSP100.4	0.002511408	0.012492156	0.20103882
RgHSP60.1	RdHSP60.16	0.004304173	0.014712911	0.292543919
RgHSP60.3	RdHSP60.14	0.00468142	0.01868424	0.250554454
RgHSP90.9	RdHSP90.1	0	0.013655361	0
