# Supplementary material for: Genome-Wide Identification and Characterization of RdHSP Genes Related to High Temperature in Rhododendron delavayi
Source: Plants (Basel). 2024 Jul 7;13(13):1878. doi: 10.3390/plants13131878 (PMC11244423; doi:10.3390/plants13131878)
Supplement: Supplementary file 1 [file plants-13-01878-s001.zip › Table S6.pdf]

Table S6 The orthologous relationships of *HSP* genes between *R. delavayi* and *R. irroratum*

Seq_1	Seq_2	Ka	Ks	Ka_Ks
RdHSP100.2	RiHSP100.6	0.107733332	1.077887371	0.099948598
RdHSP100.4	RiHSP100.4	0.16033821	0.67031236	0.239199245
RdHSP20.10	RiHSP20.4	0.002838225	0.029706854	0.095541076
RdHSP60.1	RiHSP60.10	0	0.005152446	0
RdHSP60.2	RiHSP60.9	0.001561687	0.025170147	0.062045214
RdHSP60.3	RiHSP60.8	0.184883038	0.274062981	0.674600549
RdHSP60.5	RiHSP60.11	0	0.0052678	0
RdHSP60.6	RiHSP60.12	0.043781595	0.044973801	0.973491107
RdHSP60.7	RiHSP60.16	0	0.01120469	0
RdHSP60.8	RiHSP60.1	0.00079766	0.007922609	0.100681516
RdHSP60.11	RiHSP60.14	0.000804721	0.013953891	0.057670016
RdHSP60.13	RiHSP60.6	0.008378834	0.037610432	0.222779512
RdHSP60.14	RiHSP60.20	0.009786151	0.03369839	0.290404118
RdHSP60.16	RiHSP60.18	0.008453382	0.021291348	0.397033661
RdHSP60.17	RiHSP60.17	0.00140007	0.011165074	0.125397324
RdHSP60.18	RiHSP60.5	0.014707949	0.017140187	0.858097351
RdHSP60.19	RiHSP60.4	0.002197267	0.006965994	0.315427646
RdHSP70.1	RiHSP70.17	0.026933768	0.048868294	0.551150149
RdHSP70.10	RiHSP70.15	0.031640625	1.893512225	0.016710019
RdHSP70.11	RiHSP70.29	0.062443845	0.922212357	0.067710917
RdHSP70.12	RiHSP70.2	0.003109941	0.019637843	0.158364683
RdHSP70.13	RiHSP70.15	0.038937308	1.972264423	0.019742438
RdHSP70.14	RiHSP70.24	0.001926783	0.01132097	0.170195963
RdHSP70.15	RiHSP70.25	0.005843869	0.056243403	0.103903193
RdHSP70.18	RiHSP70.28	0.001335857	0.010263588	0.130154966
RdHSP70.19	RiHSP70.29	0.002582175	0.014475721	0.178379705
RdHSP70.22	RiHSP70.16	0	0.018265743	0
RdHSP70.23	RiHSP70.12	0.100183801	0.409778422	0.244482861
RdHSP70.24	RiHSP70.11	0.030611046	0.139110648	0.220048189
RdHSP70.29	RiHSP70.7	0.061988617	0.164679621	0.37641948
RdHSP70.3	RiHSP70.4	0.043870172	0.083161328	0.527530923
RdHSP70.4	RiHSP70.6	0	0.034265571	0
RdHSP70.5	RiHSP70.19	0.026911811	0.069184821	0.388984324
RdHSP70.9	RiHSP70.23	0.011723773	0.036673304	0.319681402
RdHSP90.1	RiHSP90.2	0.001201442	0.032972343	0.036437872
RdHSP90.2	RiHSP90.11	0.028210674	0.744013475	0.037916886
RdHSP90.3	RiHSP90.9	0.001629402	0.005683639	0.286682908
RdHSP90.4	RiHSP90.10	0.002727276	0.005678261	0.480301265
RdHSP90.5	RiHSP90.11	0.001456223	0.025265547	0.057636701
RdHSP90.6	RiHSP90.3	0.015254359	0.057850702	0.263684943

RdHSP90.7	RiHSP90.4	0.014250757	0.037528581	0.37973076
RdHSP90.8	RiHSP90.5	0.001606498	0.005675575	0.283054672
