# Supplementary material for: Genome-Wide Identification and Characterization of RdHSP Genes Related to High Temperature in Rhododendron delavayi
Source: Plants (Basel). 2024 Jul 7;13(13):1878. doi: 10.3390/plants13131878 (PMC11244423; doi:10.3390/plants13131878)
Supplement: Supplementary file 1 [file plants-13-01878-s001.zip › Table S7.pdf]

Table S7 The orthologous relationships of *HSP* genes between *R. delavayi* and *R. ovatum*

Seq_1	Seq_2	Ka	Ks	Ka_Ks
RdHSP100.2	RoHSP100.2	0.011319606	0.086266506	0.131216701
RdHSP100.3	RoHSP100.10	0.005378978	0.046393044	0.115943628
RdHSP100.4	RoHSP100.9	0.161113674	0.659381927	0.244340446
RdHSP20.1	RoHSP20.3	0.071953635	0.698292901	0.103042197
RdHSP20.10	RoHSP20.9	0.017168132	0.093384711	0.183843066
RdHSP20.11	RoHSP20.19	0.015803921	0.137639909	0.114820775
RdHSP20.12	RoHSP20.8	0.024098459	0.043535536	0.553535357
RdHSP20.13	RoHSP20.6	0.019682479	0.105436463	0.186676208
RdHSP20.14	RoHSP20.7	0.013587328	0.071482635	0.190078726
RdHSP20.15	RoHSP20.4	0.031670607	0.098246965	0.322357105
RdHSP20.5	RoHSP20.20	0.168182034	0.249488249	0.674108035
RdHSP20.6	RoHSP20.2	0.024218186	0.127110953	0.190527925
RdHSP20.7	RoHSP20.11	0.027349418	0.031620608	0.864923842
RdHSP20.8	RoHSP20.17	0.019249453	0.203817508	0.094444549
RdHSP20.9	RoHSP20.15	0.054646011	0.044567565	1.226138578
RdHSP60.3	RoHSP60.18	0.208171115	0.347656807	0.598783371
RdHSP60.5	RoHSP60.21	0.01343606	0.029544301	0.454776702
RdHSP60.11	RoHSP60.5	0.001609659	0.036886866	0.043637715
RdHSP60.12	RoHSP60.2	0.020902012	0.043496177	0.480548247
RdHSP60.13	RoHSP60.19	0.010023332	0.048937889	0.204817419
RdHSP60.14	RoHSP60.15	0.0031179	0.037837341	0.082402723
RdHSP60.16	RoHSP60.3	0.007455427	0.036551334	0.203971415
RdHSP60.17	RoHSP60.9	0.009426676	0.054279626	0.173668763
RdHSP60.18	RoHSP60.1	0.017856435	0.024487056	0.729219338
RdHSP60.19	RoHSP60.16	0.006607164	0.078024008	0.084681163
RdHSP70.10	RoHSP70.24	0.019151049	1.047343811	0.018285351
RdHSP70.11	RoHSP70.20	0.005176618	0.071599043	0.0723001
RdHSP70.12	RoHSP70.2	0.001869548	0.060720941	0.030789182
RdHSP70.13	RoHSP70.24	0.000657066	0.073562177	0.008932121
RdHSP70.14	RoHSP70.21	0.004623783	0.067883981	0.068113021
RdHSP70.15	RoHSP70.31	0.011724077	0.104283914	0.112424599
RdHSP70.17	RoHSP70.32	0.074162857	0.130998503	0.566135146
RdHSP70.18	RoHSP70.27	0.039401849	0.261172945	0.150864973
RdHSP70.19	RoHSP70.20	0.063003521	0.927715539	0.067912542
RdHSP70.20	RoHSP70.22	0.004141988	0.151487983	0.027342026
RdHSP70.22	RoHSP70.8	0.001325821	0.116977545	0.011333976
RdHSP70.23	RoHSP70.12	0.088669006	0.376646649	0.235416953
RdHSP70.24	RoHSP70.11	0.029943385	0.104538884	0.28643299
RdHSP70.4	RoHSP70.23	0.00193071	0.07750339	0.024911298
RdHSP70.5	RoHSP70.18	0.039862635	0.11405193	0.349513024

RdHSP70.9	RoHSP70.16	0.018416628	0.107505521	0.171308667
RdHSP90.1	RoHSP90.5	0.003007824	0.093028626	0.032332245
RdHSP90.2	RoHSP90.2	0.027635917	0.775929564	0.035616528
RdHSP90.3	RoHSP90.8	0.003810223	0.030922254	0.123219452
RdHSP90.4	RoHSP90.4	0.010414455	0.056824949	0.183272581
RdHSP90.5	RoHSP90.2	0.003011145	0.049761634	0.060511382
RdHSP90.6	RoHSP90.7	0.033365671	0.106244259	0.314046812
RdHSP90.8	RoHSP90.1	0.00591189	0.050537248	0.116980849
