# Supplementary material for: Genome-Wide Identification and Characterization of RdHSP Genes Related to High Temperature in Rhododendron delavayi
Source: Plants (Basel). 2024 Jul 7;13(13):1878. doi: 10.3390/plants13131878 (PMC11244423; doi:10.3390/plants13131878)
Supplement: Supplementary file 1 [file plants-13-01878-s001.zip › Table S8.pdf]

Table S8 The orthologous relationships of *HSP* genes between *R. delavayi* and *R. simsii*

Seq_1	Seq_2	Ka	Ks	Ka_Ks
RdHSP20.1	RsHSP20.16	0.00536795	0.060588017	0.088597545
RdHSP20.10	RsHSP20.8	0.011407064	0.071482635	0.159578113
RdHSP20.11	RsHSP20.7	0.021128158	0.127340865	0.165918126
RdHSP20.12	RsHSP20.6	0.019231823	0.076014371	0.253002462
RdHSP20.13	RsHSP20.5	0.046707697	0.108180821	0.431755802
RdHSP20.14	RsHSP20.2	0.008130161	0.103972423	0.078195359
RdHSP20.15	RsHSP20.15	0.063560632	0.483621452	0.131426412
RdHSP20.3	RsHSP20.15	0.016729318	0.163094558	0.102574349
RdHSP20.5	RsHSP20.13	0.163916131	0.212092528	0.772851987
RdHSP20.6	RsHSP20.12	0.022772148	0.094294417	0.241500489
RdHSP20.8	RsHSP20.10	0.016491732	0.170272347	0.096855022
RdHSP20.9	RsHSP20.9	0.05442802	0.077655509	0.700890641
RdHSP60.1	RsHSP60.16	0.003989371	0.047218388	0.084487661
RdHSP60.3	RsHSP60.15	0.172021105	0.21193893	0.811654117
RdHSP60.5	RsHSP60.14	0.000797979	0.077309761	0.010321834
RdHSP60.7	RsHSP60.12	0.001638673	0.025486171	0.064296568
RdHSP60.9	RsHSP60.11	0.015085688	0.073489795	0.205275954
RdHSP60.11	RsHSP60.9	0.002415299	0.042756767	0.056489279
RdHSP60.12	RsHSP60.8	0.034284351	0.061151434	0.560646721
RdHSP60.13	RsHSP60.7	0.043727695	0.065179126	0.670884955
RdHSP60.14	RsHSP60.5	0.003332516	0.024402838	0.136562642
RdHSP60.16	RsHSP60.2	0.058173664	0.115229886	0.504848749
RdHSP60.17	RsHSP60.4	0.020542317	0.067461177	0.304505754
RdHSP70.10	RsHSP70.17	0.005636943	0.083537175	0.067478255
RdHSP70.11	RsHSP70.16	0.007014061	0.107186076	0.065438175
RdHSP70.12	RsHSP70.14	0.00248692	0.041850471	0.059423945
RdHSP70.13	RsHSP70.17	0.022130692	1.083166668	0.020431474
RdHSP70.14	RsHSP70.13	0.004505484	0.077961548	0.057791111
RdHSP70.15	RsHSP70.11	0.02965241	0.127124747	0.233254427
RdHSP70.17	RsHSP70.8	0.071115809	0.133119947	0.534223538
RdHSP70.18	RsHSP70.9	0.004688038	0.059669367	0.078566922
RdHSP70.19	RsHSP70.16	0.060623209	0.75546024	0.080246723
RdHSP70.20	RsHSP70.7	0.00691528	0.188853468	0.036617171
RdHSP70.22	RsHSP70.23	0.033342584	1.901865813	0.017531513
RdHSP70.23	RsHSP70.3	0.05272294	0.20900248	0.25225988
RdHSP70.24	RsHSP70.4	0.0375324	0.158305476	0.237088453
RdHSP70.29	RsHSP70.6	0.016247398	0.074502892	0.21807741
RdHSP70.4	RsHSP70.24	0.021445932	0.108294069	0.198034222
RdHSP70.5	RsHSP70.18	0.024821293	0.125344104	0.198025215
RdHSP70.9	RsHSP70.19	0.024467481	0.106626111	0.229469883

RdHSP90.1	RsHSP90.8	0.003612289	0.108653067	0.033246082
RdHSP90.2	RsHSP90.7	0.002423265	0.068877482	0.035182259
RdHSP90.4	RsHSP90.6	0.010409225	0.038793391	0.268324692
RdHSP90.5	RsHSP90.7	0.026426331	0.783949731	0.033709216
RdHSP90.6	RsHSP90.3	0.036190816	0.078040254	0.463745498
RdHSP90.8	RsHSP90.2	0.003756044	0.054605005	0.068785716
