# Supplementary material for: Genome-Wide Identification and Characterization of RdHSP Genes Related to High Temperature in Rhododendron delavayi
Source: Plants (Basel). 2024 Jul 7;13(13):1878. doi: 10.3390/plants13131878 (PMC11244423; doi:10.3390/plants13131878)
Supplement: Supplementary file 1 [file plants-13-01878-s001.zip › Table S9.pdf]

Table S9 The orthologous relationships of *HSP* genes between *R. delavayi* and *R. williamsianum*

Seq_1	Seq_2	Ka	Ks	Ka_Ks
RwHSP60.15	RdHSP60.11	0	0.011194238	0
RwHSP20.12	RdHSP20.10	0.002838225	0.029706854	0.095541076
RwHSP20.10	RdHSP20.5	0.209109117	0.299539547	0.698101869
RwHSP20.9	RdHSP20.6	0.003694589	0.012422644	0.297407595
RwHSP20.8	RdHSP20.3	0.027794502	0.228945903	0.12140205
RwHSP20.7	RdHSP20.1	0.008066401	0.029682357	0.271757425
RwHSP60.12	RdHSP60.2	0.083203021	0.128963859	0.645165414
RwHSP20.8	RdHSP20.11	0.115501257	1.189959289	0.0970632
RwHSP20.8	RdHSP20.15	0.056941965	0.413646688	0.137658457
RwHSP20.6	RdHSP20.3	0.061169958	0.439857037	0.139067817
RwHSP20.6	RdHSP20.9	0.261086488	0.821516218	0.31781051
RwHSP20.6	RdHSP20.11	0.129096055	1.065860263	0.121119118
RwHSP20.6	RdHSP20.15	0.005415186	0.01960896	0.276158754
RwHSP70.22	RdHSP70.15	0.047058765	0.149042456	0.315740673
RwHSP70.18	RdHSP90.3	0.015529467	0.039079921	0.397377133
RwHSP70.19	RdHSP90.4	0.000527705	0.005541897	0.095220913
RwHSP70.20	RdHSP90.5	0.008533075	0.052022499	0.164026633
RwHSP60.8	RdHSP60.7	0.003863499	0.010236571	0.377421155
RwHSP100.2	RdHSP100.2	0.104965928	1.077096479	0.09745267
RwHSP60.7	RdHSP60.14	0.011096093	0.032247958	0.34408668
RwHSP60.5	RdHSP60.16	0.033870297	0.093348523	0.362836991
RwHSP60.4	RdHSP60.17	0.009095796	0.024192203	0.375980485
RwHSP70.9	RdHSP70.4	0.059586165	0.100353958	0.59375999
RwHSP70.9	RdHSP70.14	0.088101988	0.926088186	0.095133476
RwHSP20.5	RdHSP20.11	0.007865486	0.055796441	0.140967525
RwHSP70.8	RdHSP90.8	0.002819287	0.007944464	0.354874461
RwHSP20.4	RdHSP20.12	0.009554269	0.06279817	0.152142481
RwHSP60.3	RdHSP60.13	0.055700801	0.096849873	0.575125189
RwHSP20.2	RdHSP20.9	0.031714284	0.044715227	0.709250193
RwHSP20.3	RdHSP20.7	0.015096115	0.010333114	1.460945368
RwHSP70.4	RdHSP70.12	0.002288331	0.017060037	0.134134018
RwHSP70.5	RdHSP70.11	0.012741414	0.019598284	0.650129059
RwHSP70.3	RdHSP70.19	0.075384275	0.121700415	0.619424965
RwHSP70.2	RdHSP70.18	0.028001717	0.059688176	0.46913339
RwHSP70.1	RdHSP70.17	0.03895968	0.045152776	0.862841316
