# Supplementary figures and images for: Genome-Wide Identification and Characterization of RdHSP Genes Related to High Temperature in Rhododendron delavayi
Source: Plants (Basel). 2024 Jul 7;13(13):1878. doi: 10.3390/plants13131878 (PMC11244423; doi:10.3390/plants13131878)

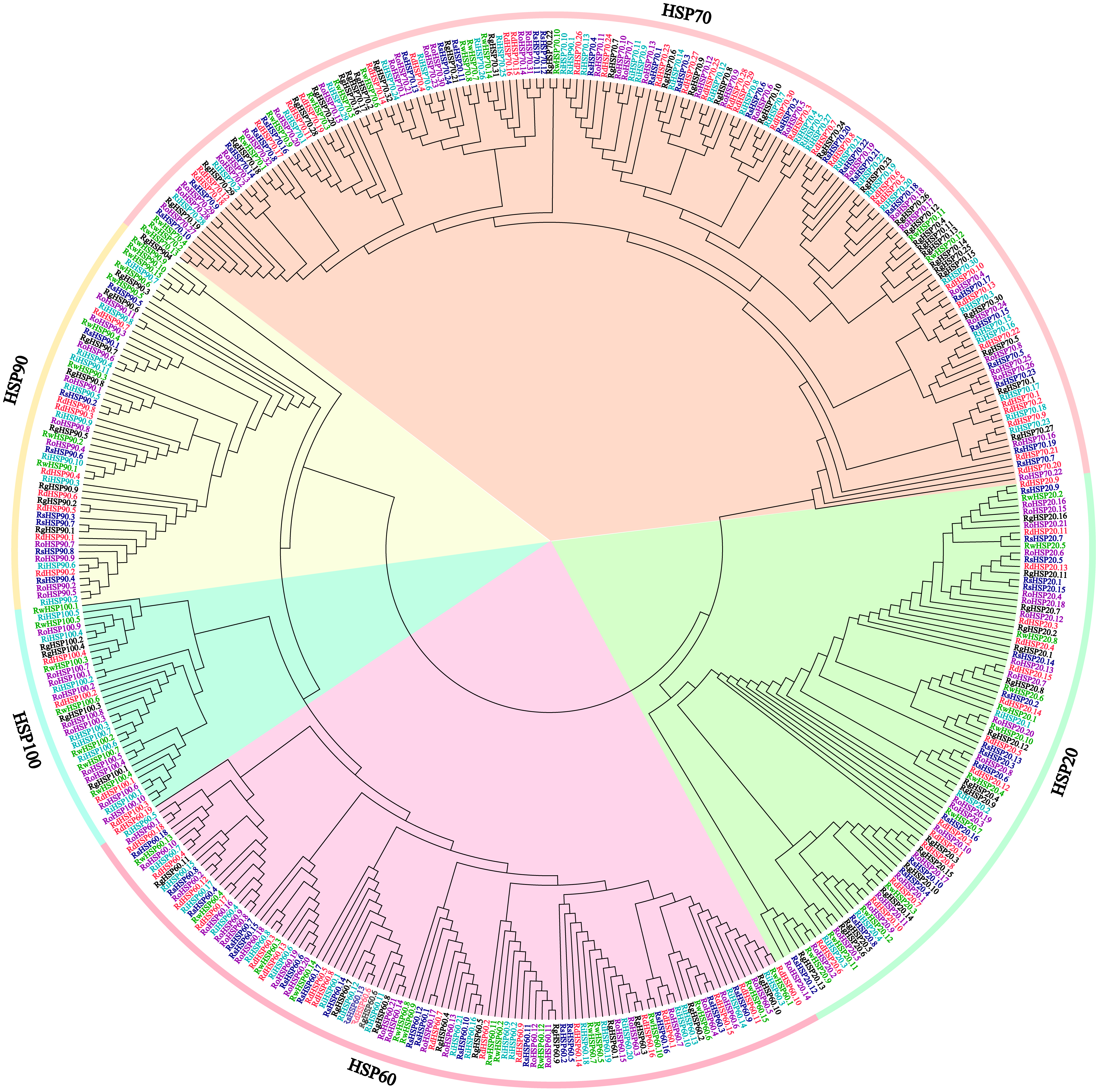

Supplement: Supplementary file 1 [file plants-13-01878-s001.zip › FIG S1.tif]
